# Coronary computed tomography angiography for clinical practice

**DOI:** 10.1007/s11604-024-01543-1

**Published:** 2024-03-08

**Authors:** Kazuki Yoshida, Yuki Tanabe, Takaaki Hosokawa, Tomoro Morikawa, Naoki Fukuyama, Yusuke Kobayashi, Takanori Kouchi, Naoto Kawaguchi, Megumi Matsuda, Tomoyuki Kido, Teruhito Kido

**Affiliations:** 1 Department of Radiology, Ehime University Graduate School of Medicine, Shitsukawa, Toon, Ehime 791-0295 Japan; 2 Department of Radiology, Matsuyama Red Cross Hospital, Bunkyocho, Matsuyama, Ehime Japan; 3 Department of Radiology, Juzen General Hospital, Kitashinmachi, Niihama, Ehime Japan

**Keywords:** Computed tomography, Coronary computed tomography angiography, Coronary artery disease, Coronary artery disease reporting and data system, Coronary artery plaque

## Abstract

Coronary artery disease (CAD) is a common condition caused by the accumulation of atherosclerotic plaques. It can be classified into stable CAD or acute coronary syndrome. Coronary computed tomography angiography (CCTA) has a high negative predictive value and is used as the first examination for diagnosing stable CAD, particularly in patients at intermediate-to-high risk. CCTA is also adopted for diagnosing acute coronary syndrome, particularly in patients at low-to-intermediate risk. Myocardial ischemia does not always co-exist with coronary artery stenosis, and the positive predictive value of CCTA for myocardial ischemia is limited. However, CCTA has overcome this limitation with recent technological advancements such as CT perfusion and CT-fractional flow reserve. In addition, CCTA can be used to assess coronary artery plaques. Thus, the indications for CCTA have expanded, leading to an increased demand for radiologists. The CAD reporting and data system (CAD-RADS) 2.0 was recently proposed for standardizing CCTA reporting. This RADS evaluates and categorizes patients based on coronary artery stenosis and the overall amount of coronary artery plaque and links this to patient management. In this review, we aimed to review the major trials and guidelines for CCTA to understand its clinical role. Furthermore, we aimed to introduce the CAD-RADS 2.0 including the assessment of coronary artery stenosis, plaque, and other key findings, and highlight the steps for CCTA reporting. Finally, we aimed to present recent research trends including the perivascular fat attenuation index, artificial intelligence, and the advancements in CT technology.

## Introduction

Coronary artery disease (CAD) is a pathophysiological condition in which atherosclerotic plaques accumulate in the coronary arteries [1]. CAD is classified into obstructive and non-obstructive CAD (invasive coronary angiography (ICA) with < 50% luminal stenosis) based on the severity of the stenosis, or stable CAD and acute coronary syndrome (ACS) based on the clinical course. Patients with stable CAD may experience chest symptoms such as chest pain on exertion owing to myocardial ischemia caused by coronary artery stenosis [1, 2]. ACS may occur in patients with both obstructive CAD and non-obstructive CAD. Lifestyle modifications, medications, and revascularization are effective measures for stabilizing or improving CAD, and severity and risk assessments of CAD are important to provide optimal treatment interventions for each patient. Coronary computed tomography angiography (CCTA) is widely used as an imaging tool for CAD assessment [1–3]. Moreover, the CAD reporting and data system (CAD-RADS) 2.0 was recently proposed for standardizing CCTA reporting [4]. In this review, we aimed to review the major trials and guidelines for CCTA to understand its clinical role. Furthermore, we aimed to introduce the CAD-RADS 2.0 including the assessment of coronary artery stenosis, plaque, and other key findings, and highlight the steps for CCTA reporting. Finally, we aimed to present recent research trends including perivascular fat attenuation index (FAI), artificial intelligence (AI), and the advancements in computed tomography (CT) technology.

## Evidence and guidelines for CCTA

### Stable CAD

CAD is a pathophysiological condition characterized by the accumulation of coronary atherosclerosis, which initially progresses asymptomatically and leads to a decrease in myocardial perfusion because of plaque progression and luminal stenosis [1]. The coronary arteries and microvasculature regulate myocardial perfusion, and the resting myocardial perfusion is maintained even at 80% luminal stenosis by dilating microvasculature [5]. As the coronary luminal stenosis worsens, the coronary artery becomes incapable of supplying adequate myocardial blood flow to meet the myocardial oxygen demand, leading to myocardial ischemia during exertion [5].

#### Diagnostic performance of CCTA

In a meta-analysis that compared the diagnostic performance of CCTA with that of exercise electrocardiography (ECG) and single photon emission computed tomography (SPECT) using ICA with ≥ 50% luminal stenosis as the reference standard, CCTA had a sensitivity of 95–99%, specificity of 68–93%, positive predictive value (PPV) of 75–93%, and negative predictive value (NPV) of 96–99%, demonstrating higher diagnostic performance than exercise ECG or SPECT [6]. Another meta-analysis reported that CCTA had a sensitivity of 97%, specificity of 78%, positive likelihood ratio (PLR) of 4.44, and negative likelihood ratio (NLR) of 0.04 when ICA ≥ 50% was used as the reference standard, and the low NLR indicates that CCTA is effective in ruling out obstructive CAD [7]. However, myocardial ischemia does not always co-exist with coronary artery stenosis. Revascularization for patients with no myocardial ischemia may worsen the prognosis; thus, the assessment of myocardial ischemia is important [8, 9]. Conventional non-invasive tools for assessing myocardial ischemia include SPECT, cardiac magnetic resonance (CMR), and positron emission tomography (PET). Additionally, fractional flow reserve (FFR) is an invasive tool measured through ICA for assessing hemodynamically significant stenosis [10]. When FFR ≤ 0.8 was used as the reference standard, CCTA demonstrated a sensitivity of 93%, specificity of 53%, PLR of 1.97, and NLR of 0.13, indicating that CCTA had inferior diagnostic performance to stress CMR, SPECT, and PET in detecting myocardial ischemia [7], while CCTA exhibited high sensitivity in detecting obstructive CAD (Table 1) [6, 7, 11–16].

**Table 1:** Diagnostic performance for obstructive CAD using CCTA

	Basis	Sensitivity (%)	Specificity (%)	PPV (%)	NPV (%)	PLR	NLR
Prospective multi-center trial							
Budoff et al. (ACCURACY) [11]	230 patients	95	83	64	99		
Meijboom et al. [12]	360 patients	99	64	86	97		
Marano et al. (NIMISCAAD) [13]	327 patients	94	88	91	91		
Arbab-Zadeh et al. (CORE-64) [14]	273 patients	91	87	90	88		
Neglia et al. (EVINCI) [15]	475 patients	91	92	83	96		
Budoff et al. (PICTURE) [16]	230 patients	92	78	82	90		
Metanalysis							
Nielsen et al. [6]	2–7 studies	95–99	68–93	75–93	96–99		
Knuuti et al. [7]	28,664 patients	97	78			4.44	0.04

*CCTA* coronary computed tomography angiography, *NLR* negative likelihood ratio, *NPV* negative predictive value, *PLR* positive likelihood ratio, *PPV* positive predictive value

A combination of CCTA and CT-derived FFR (CT-FFR) or stress CT perfusion (CTP) has been developed to overcome the inadequate diagnostic performance of CCTA in assessing myocardial ischemia [17, 18]. CT-FFR can be calculated from the CCTA data using computational fluid dynamics [19]. In a sub-study of the Prospective Comparison of Cardiac PET/CT, SPECT/CT Perfusion Imaging, and CT Coronary Angiography With Invasive Coronary Angiography (PACIFIC) trial involving patients suspected of CAD (*n* = 505 vessels), the sensitivity and specificity for diagnosing hemodynamically significant stenosis (FFR ≤ 0.8) on a per-vessel basis were 90% and 86% for CT-FFR, 68% and 83% for CCTA, 42% and 97% for SPECT, and 81% and 76% for PET [20]. The area under the curve (AUC) of CT-FFR (0.94) was significantly higher than those of CCTA (0.83), SPECT (0.70), and PET (0.87) on a per-vessel analysis, indicating higher diagnostic performance for hemodynamically significant stenosis in CT-FFR than that in CCTA [20]. CTP can also detect myocardial ischemia by acquiring images at rest and under pharmacological stress [19]. In a meta-analysis by Celeng et al., the sensitivity and specificity for diagnosing hemodynamically significant stenosis (FFR ≤ 0.8) on a per-vessel basis were 87% and 61% for CCTA alone, and 82% and 88% for CCTA plus stress CTP, respectively [18]. The combination of CCTA and CTP could improve the diagnostic performance (especially specificity) for myocardial ischemia compared with CCTA alone. Furthermore, the Perfusion Versus Fractional Flow Reserve CT Derived In Suspected Coronary (PERFECTION) study, investigating symptomatic patients with a low to intermediate pretest probability of CAD (*n* = 147), revealed that adding CTP and CT-FFR to CCTA provided incremental diagnostic value improving the specificity for identifying hemodynamically significant stenosis (FFR ≤ 0.8, ICA > 80% diameter stenosis, or total occlusion used as the reference standard) [21]. CTP or CT-FFR in combination with CCTA improves the identifications of hemodynamically significant stenosis (Table 2) [18, 21–24].

**Table 2:** Diagnostic performance for myocardial ischemia or hemodynamically significant stenosis using CTP or CT-FFR

	Basis	Method	Sensitivity (%)	Specificity (%)	PPV (%)	NPV (%)	PLR	NLR
Prospective trial								
Kitagawa et al. (AMPLIFiED study) [24]	442 vessels	CCTA	88	53	39	93		
		CCTA+CTP	73	72	47	89		
Pontone et al. (PERFECTION study) [21]	441 vessels	CCTA	99	76	61	100		
	432 vessels	CCTA+CTP	92	95	87	97		
	429 vessels	CCTA+CT-FFR	88	94	84	95		
Metanalysis								
Celeng et al. [18]	6400 vessels	CCTA	87	61			2.27	0.21
	1785 vessels	CCTA+CTP	82	88			6.97	0.21
	362 vessels	CCTA+CT-FFR	76	80			4.00	0.31
Hamon et al. [22]	5351 vessels	CCTA	86	64	53	91	2.42	0.21
	2336 vessels	CTP	82	89	79	91	7.72	0.21
	2071 vessels	CT-FFR	85	75	65	90	3.50	0.23
Pontone et al. [23]	2641 vessels	CCTA	88	64	68	87	2.39	0.17
	1036 vessels	CCTA+CTP	79	91	90	81	9.57	0.23
	1247 vessels	CT-FFR	85	75	72	80	2.82	0.22

*CCTA* coronary computed tomography angiography, *CTP* computed tomography perfusion, *CT-FFR* Computed tomography-fractional-flow reserve, *NLR* negative likelihood ratio, *NPV* negative predictive value, *PLR* positive likelihood ratio, *PPV* positive predictive value

#### Prognostic value of CCTA

The Prospective Multicenter Imaging Study for the Evaluation of Chest Pain (PROMISE) trial compared initial anatomical testing using CCTA with initial functional testing (exercise ECG, nuclear stress testing, or stress echocardiography) in symptomatic patients with suspected CAD (*n* = 10,003) [25]. No significant difference was observed in clinical outcomes (death, myocardial infarction (MI), hospitalization for unstable angina, or major procedural complications) between the CCTA-first group and functional testing first group over a median follow-up of 2.1 years (3.3% vs. 3.0%; adjusted hazard ratio [HR], 1.04 [95% confidence interval [CI]: 0.85–1.29, *p* = 0.75]). In the Scottish Computed Tomography of the Heart (SCOT-HEART) trial, which followed patients with stable chest pain (*n* = 4146) for a median of 4.8 years, the standard care plus CCTA group had lower rates of coronary deaths and non-fatal MIs than the standard care group (5-year rate 2.3% vs. 3.9%; HR 0.59 [95% CI 0.41–0.84, *p* = 0.004]), although no significant difference was observed in the frequency of invasive treatment or revascularization [26]. The Diagnostic Imaging Strategies for Patients with Stable Chest Pain and Intermediate Risk of Coronary Artery Disease (DISCHARGE) trial compared CCTA with ICA as the initial diagnostic imaging strategy in patients with intermediate pretest probability for obstructive CAD (*n* = 3561) with a median follow-up of 3.5 years [27]. The CCTA and ICA groups showed similar risks of major adverse cardiovascular events (MACE) (cardiovascular death, nonfatal MI, nonfatal stroke) (2.1% vs. 3.0%, HR 0.70 [95% CI 0.46–1.07]). In contrast, the initial CCTA strategy was associated with a lower risk of major procedure-related complications than the initial ICA strategy (0.5% vs. 1.9%, HR 0.26 [95% CI 0.13–0.55]) [27].

#### Guidelines

The 2019 European Society of Cardiology (ESC) chronic coronary syndrome guidelines recommend CCTA as Class 1/Level of evidence B when obstructive CAD cannot be clinically ruled out [1]. The 2021 AHA/ACC/ASE/CHEST/SAEM/SCCT/SCMR chest pain guidelines also recommend CCTA as Class 1/Level of evidence A for intermediate–high-risk patients with stable chest pain and no known CAD [2]. Furthermore, the 2022 Japanese Circulation Society (JCS)-focused update for stable CAD recommends CCTA as Class 1/Level of evidence A for intermediate–high-risk patients with stable chest pain [28]. This was the first guideline developed in Japan considering the results of the International Study of Comparative Health Effectiveness with Medical and Invasive Approaches (ISCHEMIA) trial. In the ISCHEMIA trial, patients with moderate to severe ischemia without left main coronary artery (LMCA) lesions were randomized to an initial invasive strategy and medical therapy or a conservative strategy. There were no significant differences in the incidence of ischemic cardiovascular events or death during a median follow-up of 3.2 years [29]. The ISCHEMIA-EXTENDED trial showed that cardiovascular mortality was lower in the initial invasive strategy compared to an initial conservative strategy; however, there was no significant difference in overall mortality between the two strategies during a median follow-up of 5.7 years [30]. Based on the ISCHEMIA trial, the 2022 JCS-focused update for stable CAD noted that the presence or absence of LMCA/LMCA equivalent is the most important aspect of reporting, as it has a significant impact on the selection of the next step of CCTA [28, 29]. If there is an LMCA/LMCA equivalent, ICA is recommended. If there is obstructive CAD other than the LMCA/LMCA equivalent, stress imaging or CT-FFR is recommended for further risk assessment and followed by a recommendation for optimized medical therapy [28]. In the context of the 2022 JCS-focused update for stable CAD, the primary objective of demonstrating myocardial ischemia is now centered on risk assessment rather than serving as the basis for revascularization.

### ACS

ACS occurs following plaque rupture and rapid thrombus occlusion and may occur in patients with both obstructive and non-obstructive CAD [31]. ACS is classified into ST-segment elevation MI (STEMI) or non-ST-segment elevation ACS (NSTE-ACS) based on the presence or absence of ECG-ST-segment elevation. NSTE-ACS is further classified into non-ST-segment elevation MI (NSTEMI) or unstable angina based on the presence or absence of myocardial cell damage [31, 32].

#### Evidence of CCTA in patients with ACS

The Rule Out Myocardial Infarction/Ischemia Using Computer-Assisted Tomography (ROMICAT-II) trial randomized patients (*n* = 1000) suspected of ACS without ischemic ECG changes or an initial positive troponin test into early CCTA or standard emergency department (ED) evaluation groups (follow-up period; 28 days) [33]. Patients in the early CCTA group had a significantly shorter average hospital stay by 7.6 h than those in the standard ED evaluation group (*p* < 0.001). In the early CCTA group, a higher proportion of patients were directly discharged from the ED compared with the standard ED evaluation group (47% vs. 12%). Moreover, no significant difference was observed in MACE (death, MI, unstable angina, and urgent coronary revascularization) within 28 days between the early CCTA or standard ED evaluation groups. The Coronary Computed Tomographic Angiography for Systematic Triage of Acute Chest Pain Patients to Treatment (CT-STAT) trial randomized low-risk patients with chest pain (*n* = 699) into early CCTA or myocardial perfusion imaging (MPI) groups [34]. The early CCTA group had significantly reduced diagnosis time than the MPI group (median, 2.9 h vs. 6.3 h; *p* < 0.0001). Moreover, no significant difference was observed in MACE (ACS, cardiac death, and revascularization) during the 6-month follow-up period in the early CCTA and MPI groups. In a recent sub-study of the Very Early Versus Deferred Invasive Evaluation Using Computerized Tomography (VERDICT) trial involving patients with ACS, CCTA demonstrated high diagnostic performance for ruling out obstructive CAD (ICA ≥ 50% stenosis) in patients with NSTE-ACS with a sensitivity of 96.5%, specificity of 72.4%, NPV of 90.9%, and PPV of 87.9% [35].

#### Guidelines

The 2020 ESC NSTE-ACS guidelines recommend CCTA instead of ICA for patients with low-to-intermediate pretest probability and negative troponin and/or inconclusive ECG findings as Class 1/Level of evidence A [32]. The 2021 AHA/ACC/ASE/CHEST/SAEM/SCCT/SCMR chest pain guidelines also recommend CCTA as Class 1/Level of evidence A for clinically negative or inconclusive ACS findings in intermediate-risk patients [2]. The 2018 JCS guidelines also recommend CCTA as Class 2A for clinically low-to-intermediate risk patients with no ECG changes and negative blood chemistry tests [3].

### MINOCA/INOCA

With the development of high-sensitivity troponin and diagnostic imaging, and the widespread use of emergency ICA for ACS, new concepts such as myocardial infarction with non-obstructive coronary arteries (MINOCA) and ischemia with non-obstructive coronary artery disease (INOCA) have been proposed [2, 32, 36, 37].

Patients with acute chest pain and positive troponin but no evidence of obstructive CAD are initially considered as working diagnosis MINOCA. However, at this stage, various pathological conditions are mixed other than MINOCA as a final diagnosis. The patient is diagnosed with MINOCA as a final diagnosis when myocardial infarction is caused by coronary disorders such as epicardial coronary spasm, microvascular spasm, microvascular dysfunction, and coronary artery dissection [32, 36]. In the MINOCA diagnostic process, CCTA is useful to exclude obstructive coronary artery disease and to diagnose spontaneous coronary artery dissection [38, 39]. In addition, when a triple rule-out scan is performed with a widened field of view, CT is useful for diagnosing pulmonary embolism and aortic dissection. Recently, CCTA could assess myocardial injury using myocardial CT late enhancement (CT-LE) and extracellular volume fraction (ECV) like MRI [40]. The addition of CT-LE/ECV to CCTA/triple-rule-out CT allows one-stop evaluation of coronary artery stenosis, aortic lesions, pulmonary embolism, and myocardial fibrosis [41]. The combination of CCTA/triple-rule-out CT and CT-LE/ECV scans might be useful for the diagnostic process for MINOCA.

INOCA is defined as (1) clinical symptoms associated with myocardial ischemia, (2) absence of obstructive CAD (< 50% diameter stenosis or FFR > 0.80), and (3) objective evidence of myocardial ischemia [36, 37]. The major mechanism of INOCA is vasospasm of epicardial coronary artery and coronary microvascular dysfunction including microvascular spasm, increase in microvascular resistance, slow flow phenomenon, and microvascular vasodilatory dysfunction [36]. In cases of suspected INOCA, CCTA would be performed to exclude significant stenosis of epicardial coronary arteries [36, 37]. JCS/CVIT/JCC 2023 Guideline Focused Update on Diagnosis and Treatment of Vasospastic Angina (Coronary Spastic Angina) and Coronary Microvascular Dysfunction recommended the consideration of CCTA as Class IIa/Level of evidence C for patients with suspected vasospastic angina [36]. At present, ^13^N-ammonia PET and stress myocardial perfusion MRI are recommended as noninvasive methods for evaluating impaired coronary microvascular function [2, 36]. Recently, Schuijf et al. reported that the combination of CCTA and CTP was useful for the diagnosis of INOCA and the combination of CCTA and CTP might be useful for the diagnostic process for INOCA [42].

These technical developments are expanding the potential applications of cardiac CT from stable CAD to ACS and MINOCA/INOCA. However, it is important to ensure its use for appropriate patients because of the limitations of cardiac CT, such as radiation exposure and risks associated with contrast agents.

## CCTA interpretation and reporting

### Images

The Society of Cardiovascular Computed Tomography (SCCT) guidelines recommend the use of axial images, multiplanar reformation (MPR), and maximum intensity projection (MIP) for reading CCTA. Curved planar reformation (CPR) is optional, and volume-rendering reformation (VR) should be considered in limited situations [43].

#### Axial image

**Fig. 1:**
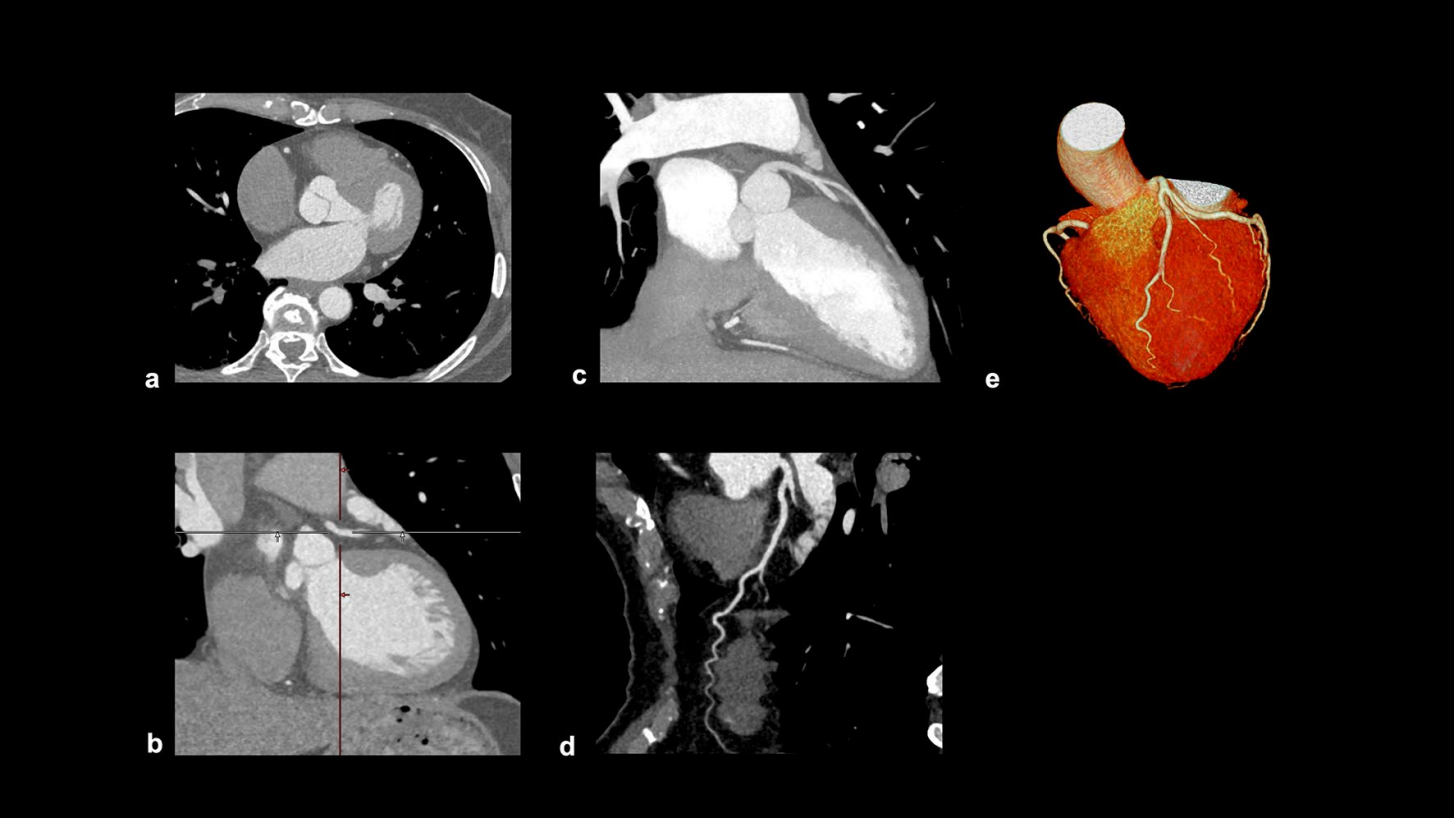
Recommended CCTA Images post-processing format. Recommended CCTA images post-processing format are as follows; **a** axial images, **b** multiplanar reformation (MPR), **c** maximum intensity projection (MIP), **d** curved planar reformation (CPR), and **e** volume rendering reformation (VR). *CCTA* coronary computed tomography angiography

Axial image is the least prone to distortion and errors caused by post-processing, and it is the basic image in reading CCTA images (Fig. 1a).

#### MPR

MPR can display coronary arteries or cardiac structures in any desired cross-section by reconstructing axial images (Fig. 1b). It is less affected by post-processing and facilitates the evaluation of cross-sections along the coronary arteries and orthogonal cross-sections. However, the slice thickness of the original image affects the image quality.

#### MIP

MIP can be reconstructed in any cross-section, similar to MPR, but it produces thicker images, making it useful for evaluating longer coronary arteries (Fig. 1c). However, MIP should not be used alone for reading CCTA because of the loss of detail caused by its thickness.

#### CPR

**Fig. 2:**
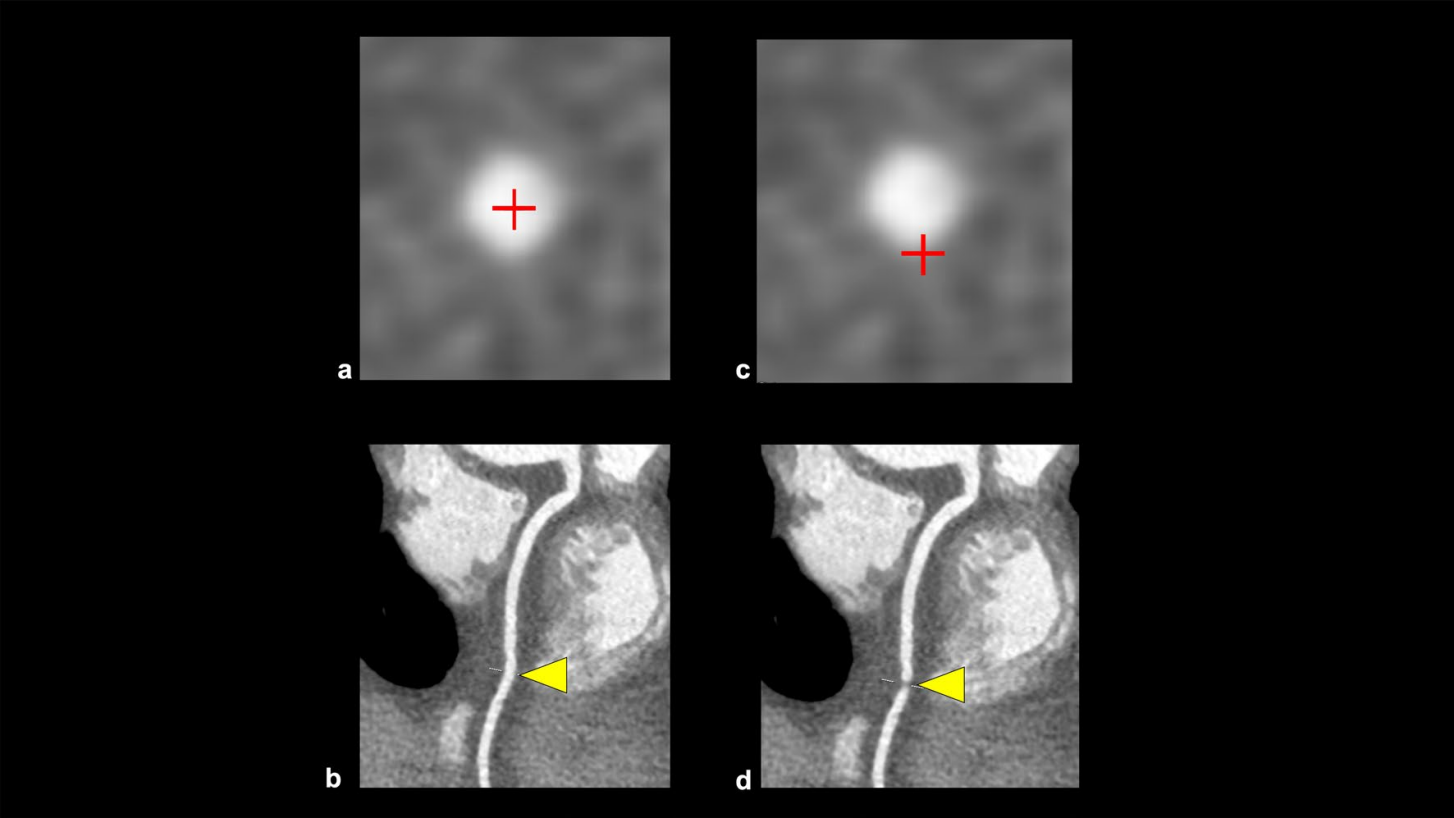
Examples of improper post-processing in curved planar reformation. The center point is properly aligned with the center of the coronary artery, and the CPR shows a coronary artery without stenosis (**a**, **b**). However, if the center point is improperly positioned, the CPR mimics coronary artery stenosis (**c**, **d**). *CPR* curved planar reformation

CPR is generated by tracing the center of a coronary artery using a workstation, and provides a single image showing the entire coronary artery (Fig. 1d). However, this image is susceptible to post-processing, and should be evaluated with cross-sectional and MPR images (Fig. 2).

#### VR

VR is not used for evaluating coronary artery stenosis, but it is useful for visualizing the three-dimensional morphology of coronary arteries (Fig. 1e).

### CAD-RADS

CAD-RADS is a scoring system for coronary artery stenosis, with recommendations for additional testing and patient management depending on the category [44]. The 2021 Expert Consensus by the SCCT recommends using CAD-RADS for reporting CCTA findings [45]. In 2022, CAD-RADS was updated to version 2.0, which introduced additional categories for plaque amount, revised management considerations, and revisions to modifiers [4]. CAD-RADS 2.0 is valuable for not only standardizing CCTA readings and reporting but also supporting patient management. However, both radiologists and attending physicians must properly understand the content and usefulness of CAD-RADS to be widely adopted and utilized effectively. Therefore, we outline CAD-RADS2.0 below.

#### Stenosis evaluation

**Fig. 3:**
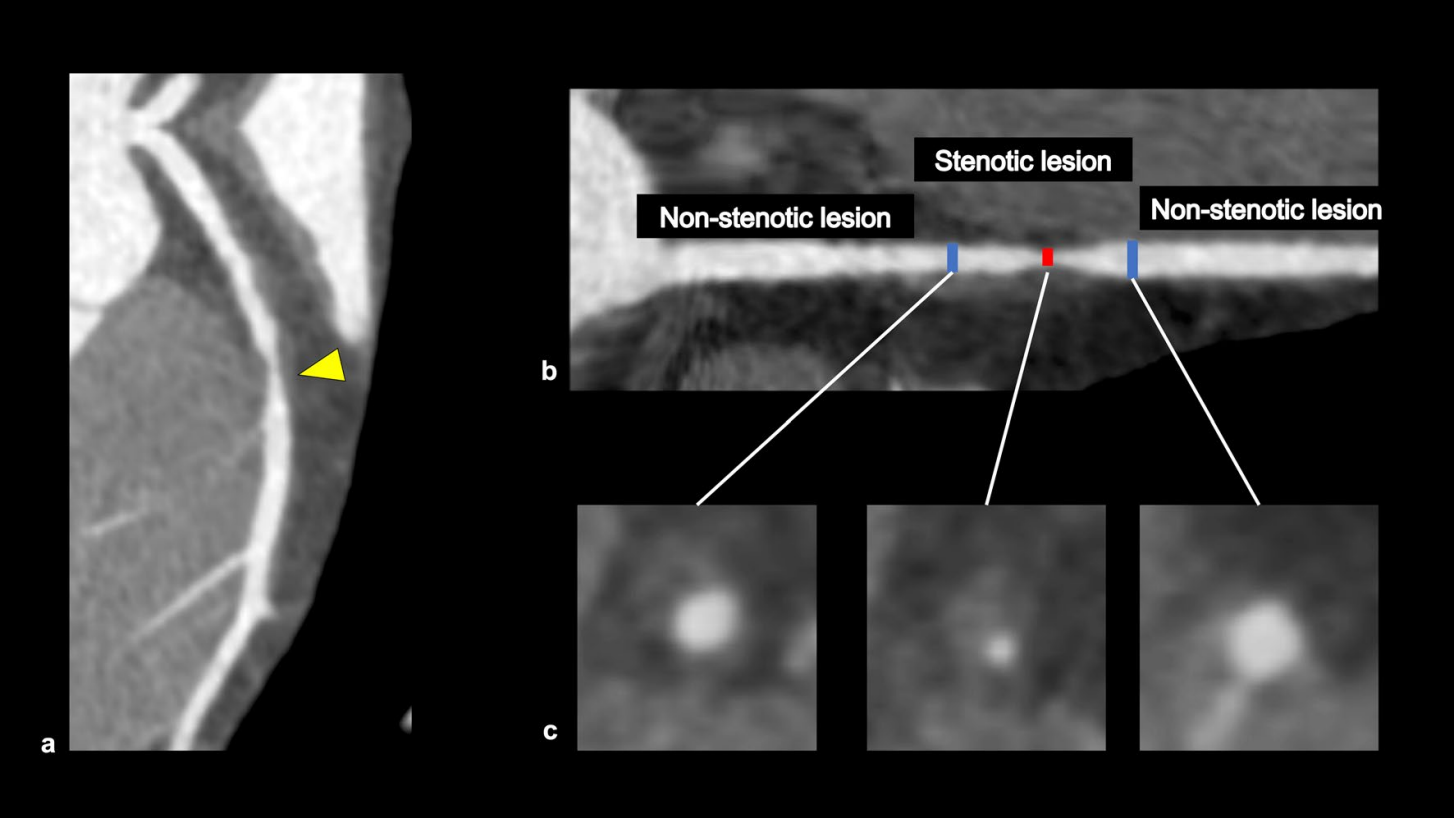
Coronary artery stenosis assessment. A CPR image shows moderate stenosis in the middle portion of the LAD (**a**). The severity of coronary artery stenosis is semi-quantitatively determined by comparing the most stenotic lesions with nearby non-stenotic coronary arteries (proximal and distal) (**b**: straightened multiplanar reformat image, **c**: cross-sectional image). *CPR* curved planar reformation, *LAD* left anterior descending artery

**Fig. 4:**
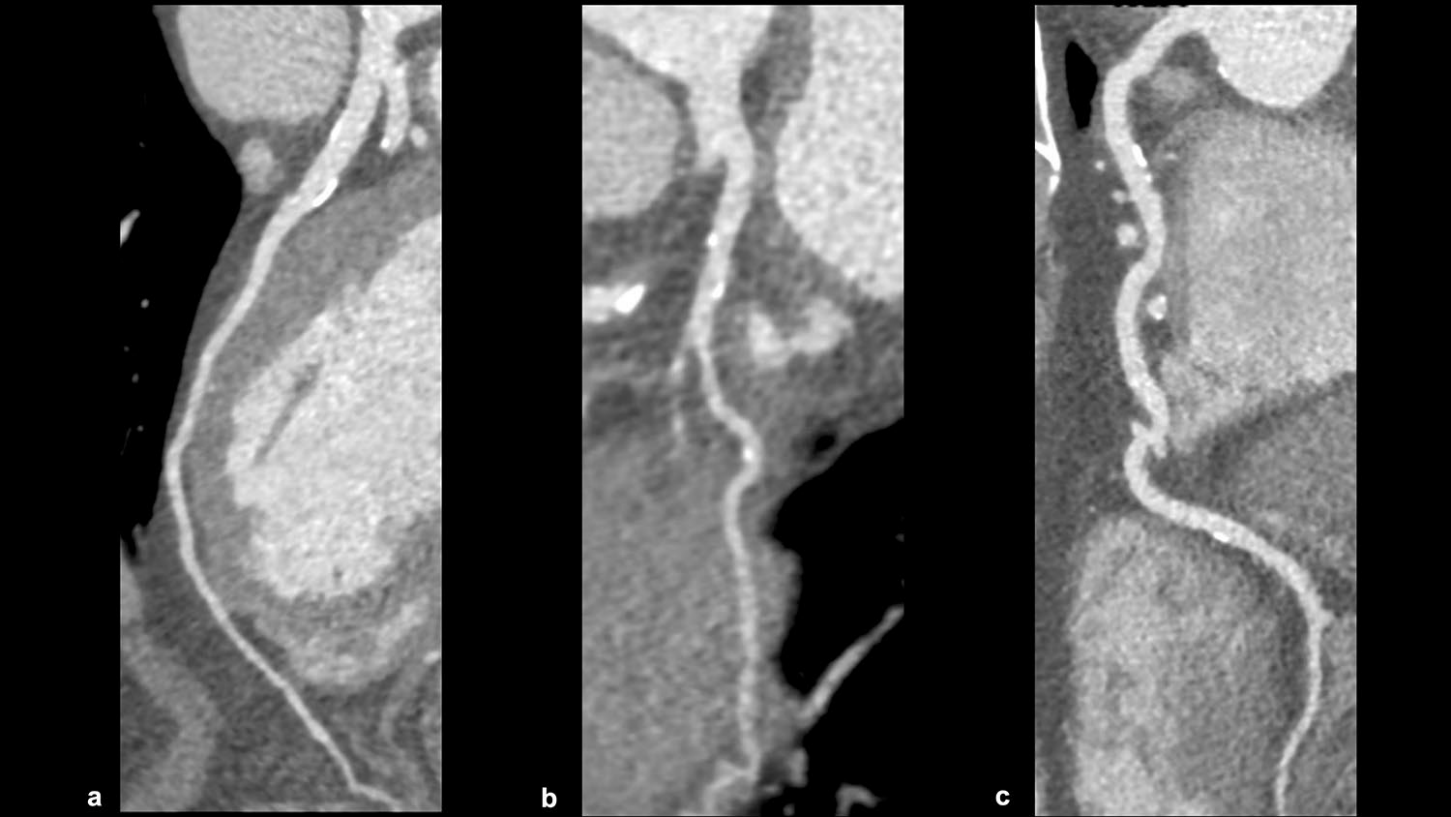
CAD-RADS 1/P3 (stable chest pain). A CPR image shows minimal stenosis of the coronary arteries (**a**: LAD, **b**: LCX, and **c**: RCA), and this patient has severe amounts of coronary plaque (CACS = 440). According to the CAD-RADS 2.0, this case is categorized as CAD-RADS 1/P3. *CPR* curved planar reformation, *LAD* left anterior descending artery, *LCX* left circumflex artery, *RCA* right coronary artery, *CACS* coronary artery calcium score

**Fig. 5:**
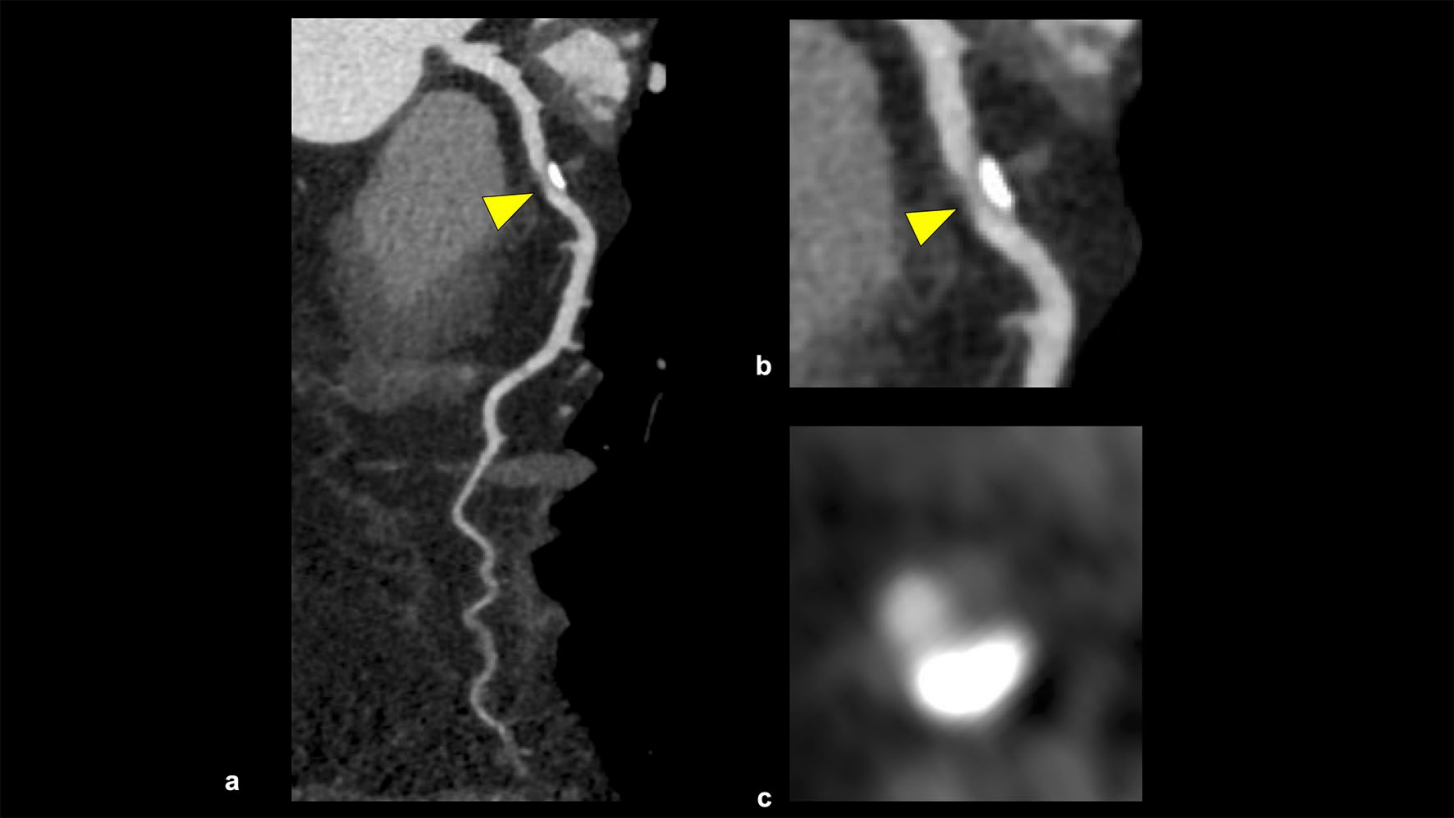
CAD-RADS 3/P2 (stable chest pain). CPR and cross-sectional images show moderate stenosis in the proximal portion of the LAD (**a**–**c**), and this patient has moderate amounts of coronary plaque (CACS = 156). According to CAD-RADS 2.0, this case is categorized as CAD-RADS 3/P2. *CPR* curved planar reformation, *LAD* left anterior descending artery, *CACS* coronary artery calcium score

**Fig. 6:**
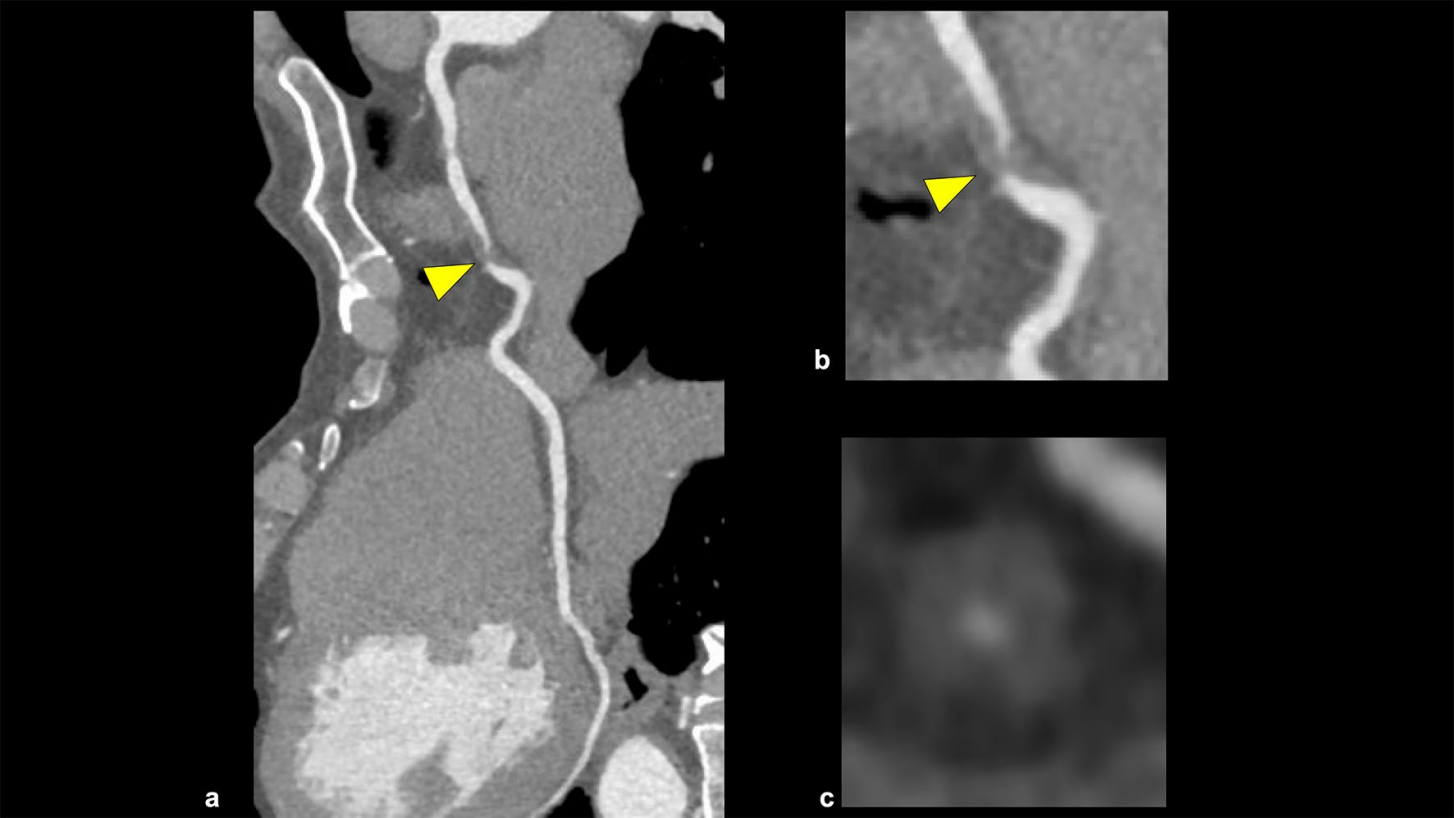
CAD-RADS 4A/P2 (stable chest pain). CPR and cross-sectional images show severe stenosis in the mid portion of the RCA (**a**–**c**), and this patient has moderate amounts of coronary plaque (SIS = 3). According to CAD-RADS 2.0, this case is categorized as CAD-RADS 4A/P2. *CPR* curved planar reformation, *RCA* right coronary artery, *CACS* coronary artery calcium score, *SIS* segment involvement score

This category in CAD-RADS 2.0 is classified based on the most stenotic lesion (target for evaluation: vessels with diameter > 1.5 mm) at the patient level [4]. The severity of coronary artery stenosis is semi-quantitatively determined by comparing the most stenotic lesions with nearby non-stenotic coronary arteries (proximal and distal) (Fig. 3). The categories in CAD-RADS 2.0 are as follows: 0 (no visible stenosis), 1 (1–24% minimal stenosis) (Fig. 4), 2 (25–49% mild stenosis), 3 (50–69% moderate stenosis) (Fig. 5), 4A (70–99% severe stenosis) (Fig. 6), 4B (left main stenosis > 50% or three-vessel 70–99% severe stenosis), and 5 (100% total occlusion). CAD-RADS 2.0 also includes statements associated with further cardiac investigations and management considerations. Moreover, identifying whether a patient falls into CAD-RADS 3 or higher is clinically important because they may require additional testing and treatment (Tables 3, 4) [4]. The presence or absence of LMCA/LMCA equivalent is also an important aspect of reporting, as it has a significant impact on the selection of the next step of CCTA [4].

**Table 3:** CAD-RADS reporting and data system for patients presenting with stable chest pain. Reprinted with permission of Elsevier from Cury et al. [4]

Category	Degree of maximal coronary stenosis	Interpretation	Further cardiac investigation	Management considerations
CAD-RADS 0	0% (no plaque or stenosis)	Absence of CAD^a^	None	– Reassurance. Consider non-atherosclerotic causes of symptoms
CAD-RADS 1	1–24% (minimal stenosis or plaque with no stenosis^b^)	Minimal non-obstructive CAD^b^	None	– Consider non-atherosclerotic causes of symptoms – P1: consider risk factor modification and preventive pharmacotherapy – P2: risk factor modification and preventive pharmacotherapy – P3 or P4: aggressive risk factor modification and preventive pharmacotherapy
CAD-RADS 2	25–49% (mild stenosis)	Mild non-obstructive CAD	None	– Consider non-atherosclerotic causes of symptoms – P1 or P2: risk factor modification and preventive pharmacotherapy – P3 or P4: aggressive risk factor modification and preventive pharmacotherapy
CAD-RADS 3	50–69%	Moderate stenosis	Consider functional assessment^c^	– P1, P2, P3 or P4: aggressive risk factor modification and preventive pharmacotherapy – Other treatments (including anti-anginal therapy) should be considered per guideline-directed care^d^ – When modifier I+, consider ICA, especially if frequent symptoms persist after guideline-directed medical therapy
CAD-RADS 4	A: 70–99% stenosis or B: left main ≥ 50% or 3—vessel obstructive (≥ 70%) disease	Severe stenosis	A: consider ICA^e^ or functional assessment B: ICA is recommended	P1, P2, P3 or P4: aggressive risk factor modification and preventive pharmacotherapy – Other treatments (including anti-anginal therapy and options of revascularization) should be considered per guideline directed care^c^
CAD-RADS 5	100% (total occlusion)	Total coronary occlusion or sub-total occlusion	Consider ICA, functional, and/or viability assessment	P1, P2, P3 or P4: aggressive risk factor modification and preventive pharmacotherapy – Other treatments (including anti-anginal therapy and options of revascularization) should be considered per guideline-directed care^c^
CAD-RADS N	Non-diagnostic study	Obstructive CAD cannot be excluded	Additional/alternative evaluation may be needed	

The CAD-RADS classification should be applied on a per-patient basis for the clinically most relevant (usually highest-grade) stenosis. All vessels greater than 1.5 mm in diameter should be graded for stenosis severity. CAD-RADS will not apply for smaller vessels (< 1.5 mm in diameter)

^a^CAD: coronary artery disease

^b^CAD-RADS 1—this category should also include the presence of plaque with positive remodeling and no evidence of stenosis

^c^Functional assessment includes CT-FFR, CTP, stress testing (ETT, stress echocardiogram, SPECT, PET, cardiac MRI) or invasive FFR

^d^Guideline-directed care per 2021 AHA/ACC chest pain guidelines, 2012 ACC/AHA guideline for the diagnosis and management of patients with stable ischemic heart disease and 2019 ACC/AHA prevention guidelines. Further evaluation of CAD-RADS 3 and 4A with functional imaging or invasive coronary angiography should be considered to identify a target lesion (if unknown) and if the patient has persistent symptoms despite adequate medical therapy

^e^ICA—invasive coronary angiography may be favored if high-grade stenosis (> 90%), high-risk plaque features or I þ (presence of lesion-specific ischemia on CT FFR or perfusion defects by CTP) or concordant ischemia by other stress tests and a candidate for revascularization. It should be clarified that the benefit of revascularization should be confined to patients with persistent symptoms despite optimal medical therapy

**Table 4:** CAD-RADS reporting and data system for patients presenting with acute chest pain. Reprinted with permission of Elsevier from Cury et al. [4]

Category	Degree of maximal coronary stenosis	Interpretation	Cardiac investigation	Management considerations
CAD-RADS 0	0%	ACS highly unlikely	– No further evaluation of ACS is required – If Tn (+) consider other sources of increased troponin	– Reassurance
CAD-RADS 1	1–24%^a^	ACS unlikely	– No further evaluation of ACS is required – If Tn (+) consider other sources of increased troponin	– P1 or P2: referral for outpatient follow-up for risk factor modification and preventive pharmacotherapy – P3 or P4: referral for outpatient follow-up for aggressive risk factor modification and preventive pharmacotherapy
CAD-RADS 2	25–49%	ACS less likely	– No further evaluation of ACS is required – If clinical suspicion of ACS is high, Tn (+) or HRP features, consider hospital admission with cardiology consultation	– P1 or P2: referral for outpatient follow-up for risk factor modification and preventive pharmacotherapy – P3 or P4: referral for outpatient follow-up for aggressive risk factor modification and preventive pharmacotherapy
CAD-RADS 3	50–69%	ACS possible	– Consider hospital admission with cardiology consultation – Consider functional assessment^b^	– P1, P2, P3 or P4: preventive management, including aggressive preventive pharmacotherapy. Other treatments, including anti-anginal therapies, should be considered per guideline-directed care^c^ – When modifier I+, consider ICA
CAD-RADS 4	A: 70–99% or B: left main ≥ 50% or 3-VD	ACS likely	– Hospital admission with cardiology consultation A: consider ICA^d^ or functional assessment B: ICA is recommended	– P1, P2, P3 or P4: preventive management, including aggressive preventive pharmacotherapy – Other treatments, including anti-anginal therapies and options of revascularization, should be considered per guideline-directed care^c^
CAD-RADS 5	100% (total occlusion)	ACS very likely	– Hospital admission with cardiology consultation. Expedited ICA and revascularization if suspected acute occlusion^e^	– P1, P2, P3 or P4: preventive management, including aggressive preventive pharmacotherapy – Other treatments (including anti-anginal therapies and options of revascularization) should be considered per guideline-directed care^c^
CAD-RADS N	Non-diagnostic study	ACS cannot be excluded	Additional or alternative evaluation for ACS is needed	

The CAD-RADS classification should be applied on a per-patient basis for the clinically most relevant (usually highest-grade) stenosis. All vessels greater than 1.5 mm in diameter should be graded for stenosis severity. CAD-RADS will not apply for smaller vessels (< 1.5 mm in diameter)

^a^CAD-RADS 1—this category should also include the presence of plaque with positive remodeling and no evidence of stenosis

^b^Functional assessment includes CT-FFR, CTP, stress testing (ETT, stress echocardiogram, SPECT, PET, Cardiac MRI) or invasive FFR

^c^Guideline-directed care per 2021 AHA/ACC chest pain guidelines, 2012 ACC/AHA guideline for the diagnosis and management of patients with stable ischemic heart disease and 2019 ACC/AHA prevention guidelines

^d^ICA—invasive coronary angiography. It should be clarified that benefit of revascularization is confined to patients with persistent symptoms despite optimal medical therapy

^e^Unless the total coronary occlusion can be identified as chronic (through CT and clinical characteristics or patient history)

#### Plaque evaluation

The assessment of coronary artery plaque amount is newly recommended in CAD-RADS 2.0. Based on the overall amount of coronary plaque, the plaque category is classified into four levels: P1 (mild), P2 (moderate), P3 (severe), and P4 (extensive). This classification is based on three methods: coronary artery calcium score (CACS), segment involvement score (SIS), or visual assessment (Table 5) [4]. CACS is a traditional and reproducible method that assesses the total amount of calcified plaque [46]. It is useful for CAD risk stratification but underestimates non-calcified plaque. SIS is calculated by the sum of calcified and non-calcified segments for every 16 coronary segments (with a maximum score of 16) and is associated with cardiovascular outcome [47]. Visual assessment is based on the number of involved vessels and plaque amount. The most severe findings among these methods are used to assess the plaque category in CAD-RADS 2.0 [4].

**Table 5:** P category in CAD-RADS version 2.0. Reprinted with permission of Elsevier from Cury et al. [4]

Category	Overall amount of coronary plaque	CAC	SIS^a^	Visual^a^
P1	Mild	1–100	≤ 2	1–2 vessels with mild amount of plaque
P2	Moderate	101–300	3–4	1 -2 vessels with moderate amount; 3 vessels with mild amount of plaque
P3	Severe	301–999	5–7	3 vessels with moderate amount; 1 vessel with severe amount of plaque
P4	Extensive	≥ 1000	≥ 8	2–3 vessels with severe amount of plaque

Categories may not always correspond across different scores; if discrepant use CAC = coronary artery calcium or total plaque burden quantification, if available. SIS = Segment Involvement Score

^a^Please note that CAD-RADS 0 denotes the absence of stenosis or plaque, therefore P0 is not required as a classification. As there is currently no one single method that should be used to identify the overall amount of plaque, CAD-RADS recommends that imagers select the technique which is considered most appropriate at a given institution

#### Modifiers

**Fig. 7:**
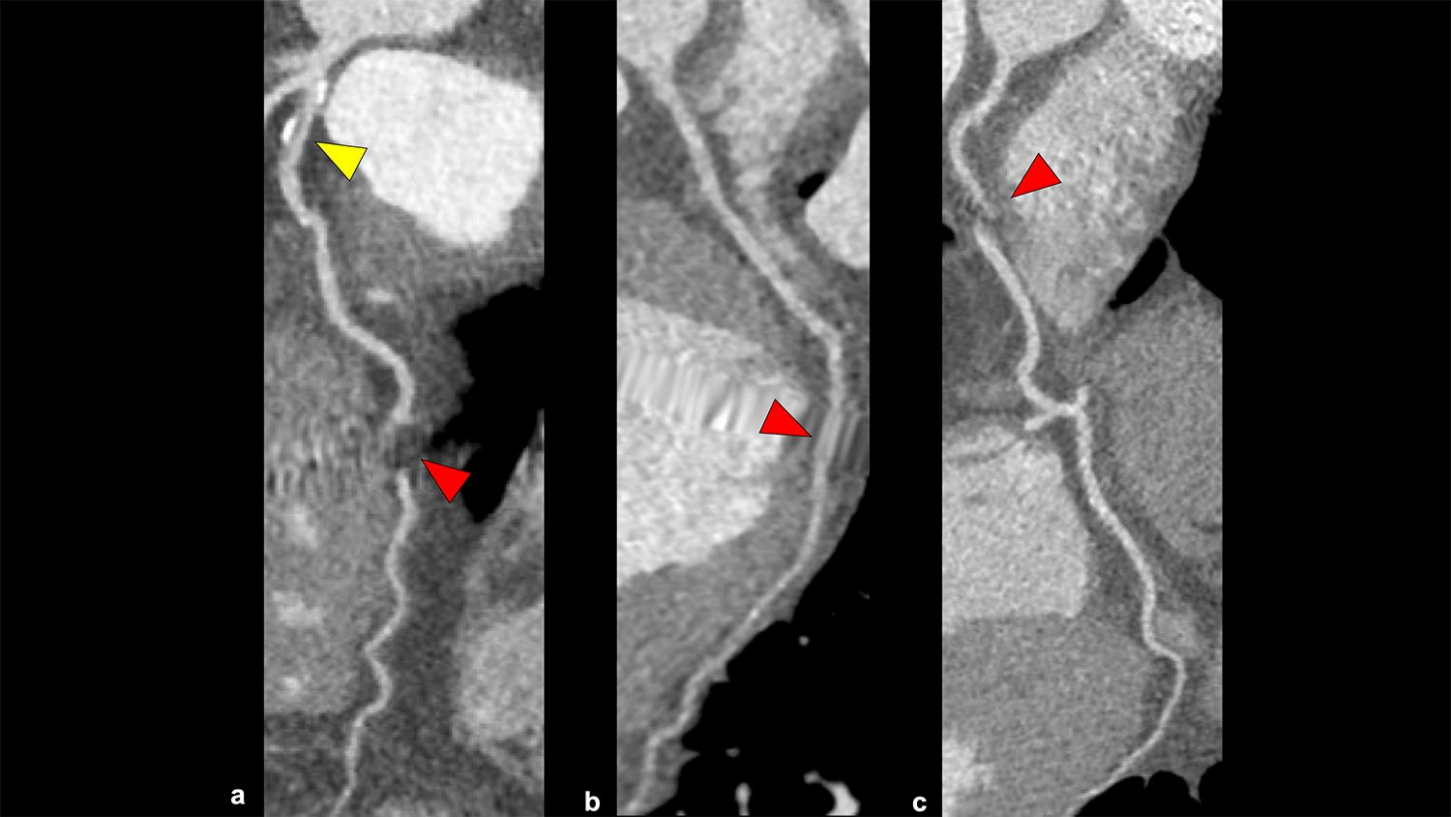
CAD-RADS 3/P2/N (stable chest pain). A CPR image shows moderate stenosis in the proximal portion of the LAD artery (**a**: yellow arrowhead), and this patient has moderate amounts of coronary plaque (CACS = 119). However, motion artifacts impair the accuracy of coronary artery assessment in certain portions (**a**–**c**: red arrowhead). According to CAD-RADS 2.0, this case is categorized as CAD-RADS 3/P2/N. *CPR* curved planar reformation, *LAD* left anterior descending artery, *CACS* coronary artery calcium score

Various modifiers are sometimes used in addition to CAD-RADS to provide further information. The following modifiers are used: non-diagnostic (N), stent (S), graft (G), high-risk plaque (HRP), ischemia (I), and exception (E). Modifier N is added when a non-diagnostic lesion is present because of calcium blooming or artifacts (Fig. 7). Modifier I is added based on CT-FFR or CTP findings and is classified as “I+” (ischemia positive), “I−” (ischemia negative), or “I±” (borderline or indeterminate) (Table 6) [4]. Modifier E is used to represent non-atherosclerotic causes of coronary artery abnormalities such as anomalous origin of the coronary arteries, coronary artery aneurysm, coronary artery fistula, extrinsic coronary artery compression, and arterio-venous malformation. Modifier S, G, and HRP categories are discussed in a later paragraph.

**Table 6:** Modifier I in CAD-RADS version 2.0

	CT-FFR	CTP		
		Stress CTP	Rest CTP	Interpretation
I+	Abnormal ≦ 0.75 >> Consider ICA for individuals likely to benefit from revascularization	Perfusion defect (+)	Negative (−)	– Reversible perfusion defect – Myocardial ischemia
		Perfusion defect (+)	Perfusion defect (+) * It is smaller than stress CTP perfusion defect	– Peri-reversible perfusion defect – Peri-infarct ischemia (ischemia + infarction)
I−	Normal > 0.80 >> Defer ICA and optimize medical therapy	Perfusion defect (+)	Perfusion defect (+) * It is equal to the extent of stress CTP perfusion defect	– Fixed-perfusion defect – Myocardial infarct without ischemia
		Negative (−)	Negative (−)	– No perfusion defects – No ischemia
I±	Borderline 0.76–0.80 >> Consider ICA based on symptoms, lesion location, and trans-lesional pressure loss and for individuals likely to benefit from revascularization	The presence of ischemia is borderline or unclear		

*CT-FFR* computed tomography-fractional-flow reserve, *CTP* CT perfusion, *ICA* invasive angiography

### Artifacts on CCTA

CCTA is vulnerable to artifacts, and understanding these artifacts on CCTA is important for optimal CCTA reporting. This section will introduce representative artifacts observed on CCTA including motion artifacts, blooming effect, beam hardening artifacts, and banding artifacts.

#### Motion artifacts

**Fig. 8:**
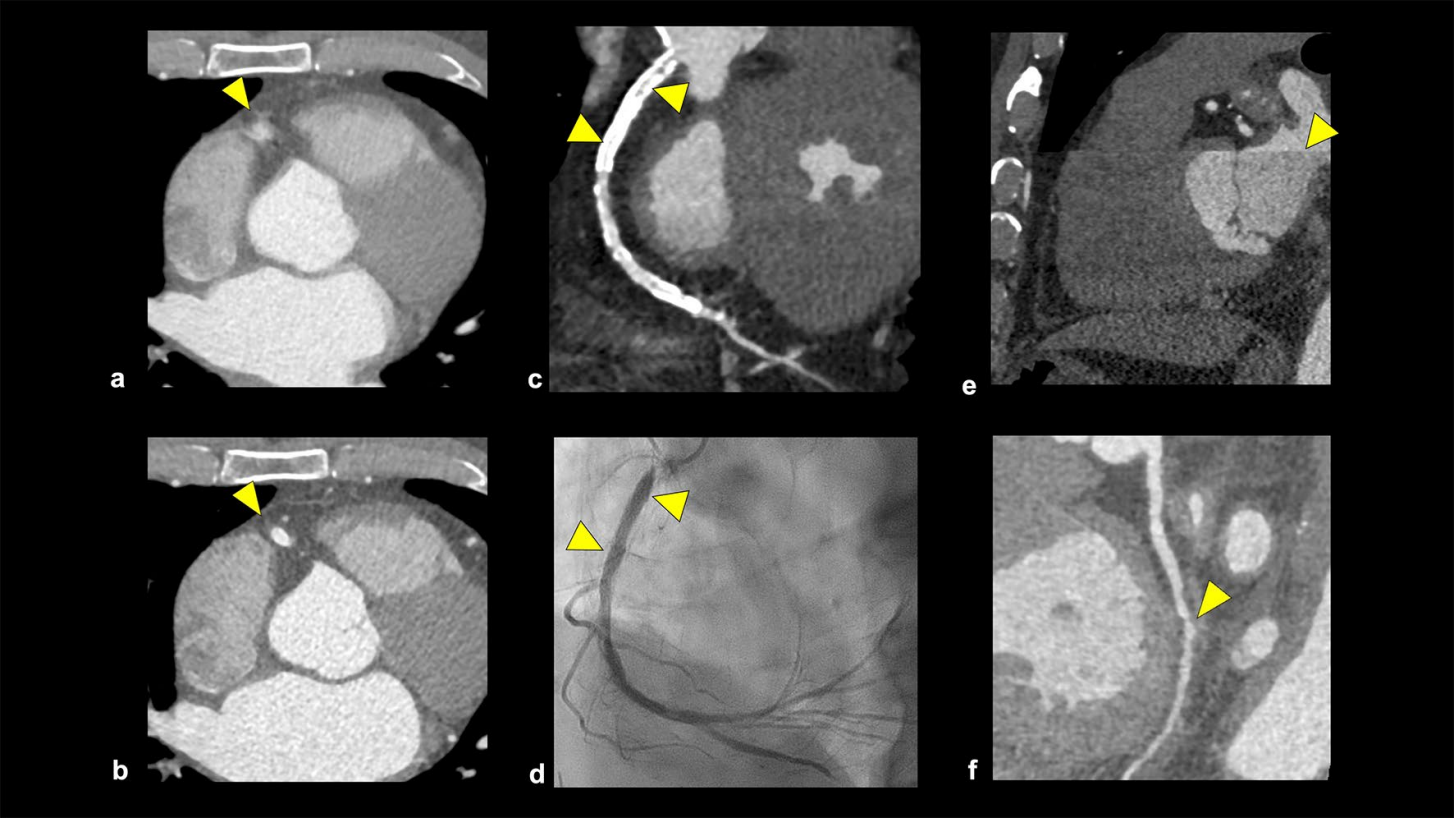
Overview of artifacts in CCTA. A motion artifact impairs the accuracy of coronary artery assessment (**a**: arrowhead), and the adjustment of reconstruction cardiac phases in the post-processing can reduce the motion artifact (**b**: arrowhead). Beam hardening artifact and blooming effect derived from coronary stent are observed in the proximal portion of the RCA (**c**: arrowhead), but ICA shows no evidence of in-stent restenosis (**d**). A banding artifact is observed on the MPR image (**e**: arrowhead), and mimics coronary artery stenosis on the CPR image (**f**: arrowhead). *RCA* right coronary artery, *ICA* invasive angiography, *MPR* multiplanar reconstruction, *CPR* curved planar reformation

Motion artifacts are caused by high heart rates, irregular heart rates, and inadequate breath-holding. Using beta-blockers, multi-segment reconstruction, or adjusting the reconstruction phase placement can be used to address these motion artifacts (Fig. 8a, b) [48, 49].

#### Blooming effect and beam hardening artifacts

The partial volume effect can cause calcified plaques and coronary stents to appear larger than their actual size, known as the blooming effect, which leads to an overestimation of coronary stenosis. In post-processing, the blooming effect can be reduced by using a sharp kernel or iterative reconstruction [49, 50]. Beam hardening artifacts sometimes occur as low attenuation areas around severely calcified plaques, iodine-contrast materials, or stents, which may be misinterpreted as stenosis. Higher tube-voltage scan, contrast agent protocol modification, and virtual monoenergetic images through dual-energy CT can reduce beam hardening artifacts (Fig. 8c, d) [49, 51].

#### Banding artifacts

Banding artifacts occur when a patient’s heart rate changes during a scan, especially in patients with high heart rates or arrhythmias. This can be avoided by shortening the scan time, for example, using wide-detector CT or high-pitch helical-mode dual-source CT (Fig. 8e, f) [49].

## Post-stenting/post-coronary artery bypass grafting CCTA

**Fig. 9:**
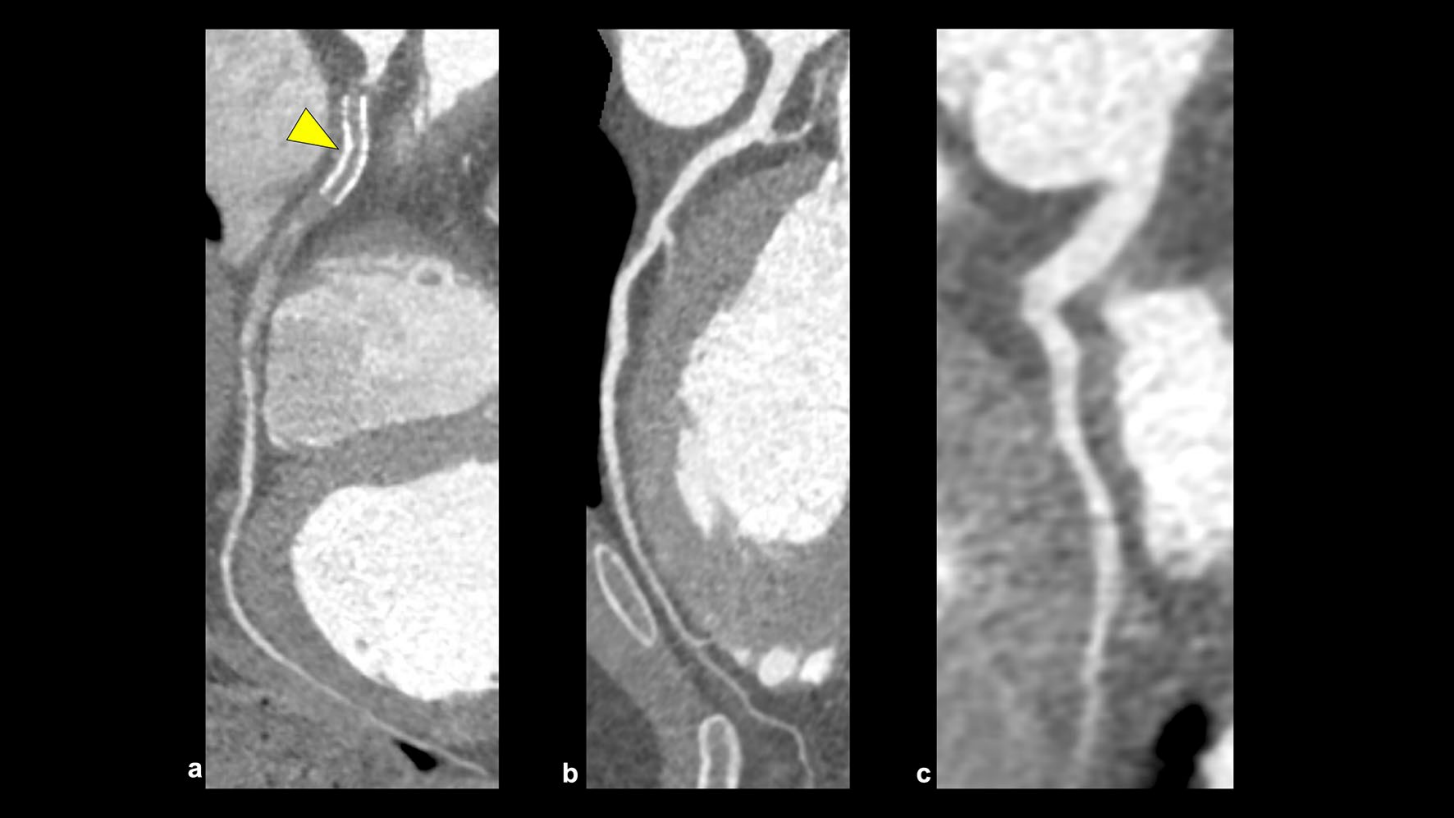
Modifier “S” in CAD-RADS 2.0. A CPR image shows in-stent restenosis in the proximal portion of the RCA (**a**: arrowhead), and this patient has severe amounts of coronary plaque. No significant stenosis is observed in the LAD or LCX (**b**, **c**). According to CAD-RADS 2.0, the patient is classified as CAD-RADS 5/P3/S. *CPR*; curved planar reformation, *RCA* right coronary artery, *LAD* left anterior descending artery, *LCX* left circumflex artery

**Fig. 10:**
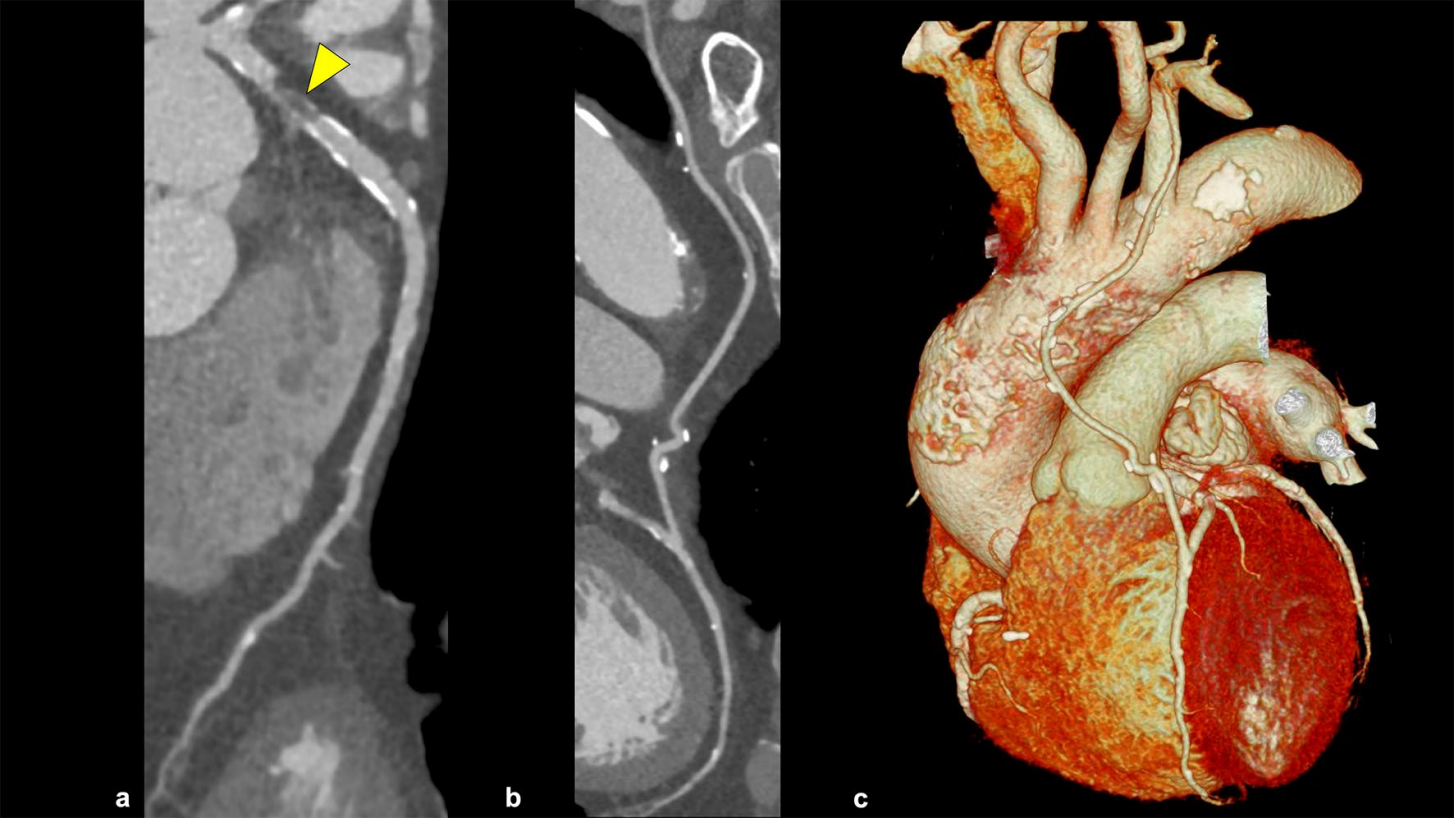
Modifier “G” in CAD-RADS 2.0. CPR images show a severe stenosis in the proximal portion of the LAD (**a**; arrowhead), and a patent LIMA graft to the LAD (**b**). This patient has a severe amount of coronary plaque. The native coronary artery proximal to the graft anastomoses should not be evaluated for CAD-RADS coding when evaluating the CCTA of patients with CABG. According to CAD-RADS 2.0, this case is classified as CAD-RADS 1/P3/G. *CPR* curved planar reformation, *LAD* left anterior descending artery, *LIMA* left internal mammary artery, *CABG* coronary artery bypass grafting

CCTA is a useful non-invasive tool for assessing coronary arteries in patients with post-stenting or coronary artery bypass grafting (CABG). Modifier S or G is added to CAD-RADS 2.0 in these cases (Figs. 9 and 10). In addition, when assessing CABG using CCTA, it is important to know the type of bypass graft prior to the CCTA scan and reading.

### Post-stenting CCTA

A recent meta-analysis of 35 studies (involving 4131 stents in 2656 patients) reported that CCTA exhibited a sensitivity of 90%, specificity of 94%, PLR of 14.0, and NLR of 0.10 for identifying in-stent restenosis [52]. However, the diagnostic performance was impaired under certain conditions such as stents with strut thickness ≥ 100 μm, stent diameter < 3.0 mm, heart rate ≥ 65 beats per minute, and bifurcated stents [52]. The technical challenge for assessing small-diameter stents is spatial resolution [55]. Recently, new-generation CT hardware such as ultra-high spatial resolution CT (UHR-CT) and photon-counting detector CT (PCD-CT) can improve spatial resolution and stent lumen visualization [53]. Furthermore, a super-resolution deep learning reconstruction technique has been developed which improves the small-diameter stent lumen assessment [54–56]. In this way, technical challenges remain in the evaluation of small-diameter stents, but it is expected that advances in hardware and software will overcome these limitations.

### Post-CABG CCTA

A recent meta-analysis evaluating 2482 bypass grafts from 959 patients demonstrated that CCTA had a sensitivity of 98%, specificity of 98%, and AUC of 0.99 for detecting CABG stenosis > 50% [57]. In patients with post-CABG, CCTA is associated with higher radiation exposure due to its wide scan range, but recent technological advancements have emerged to solve this issue. Whole-heart coverage CT scanners can assess bypass graft patency and native coronary artery stenoses with lower radiation exposure [58]. While CCTA demonstrates high diagnostic performance for evaluating graft patency, it is important to note its limitation that CCTA cannot assess the directionality of blood flow within the graft.

## Coronary atherosclerotic plaque assessment

### Importance of plaque assessment

**Fig. 11:**
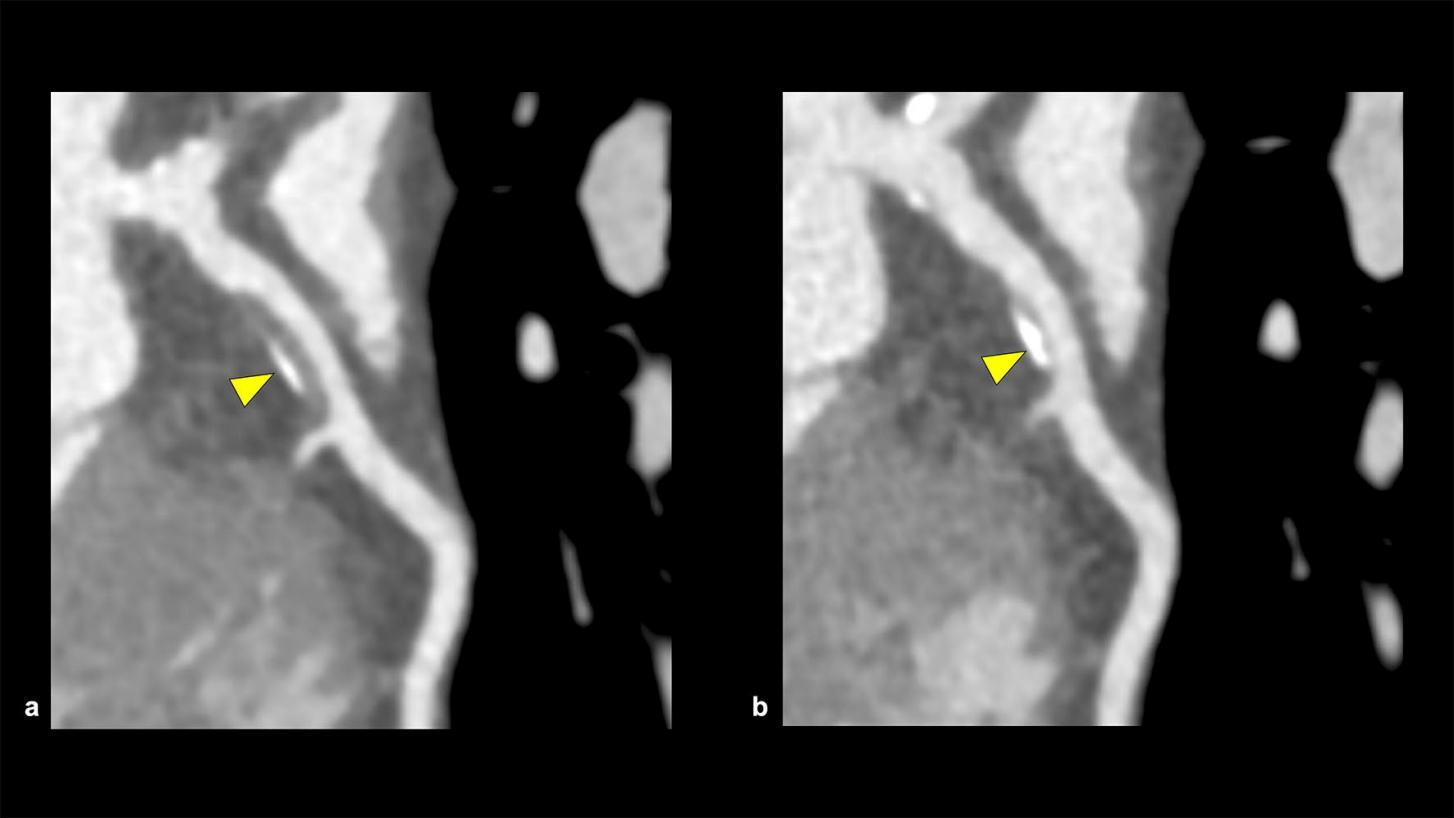
Characteristics change in coronary artery plaque on CCTA. A CPR image shows a partially calcified plaque in the proximal portion of the LAD in the first CCTA (**a**: arrowhead). After 3 years of optimal medication therapy, the coronary plaque was downsized and the calcified portion increased in the second CCTA (**b**: arrowhead). *CPR* curved planar reformation, *LAD* left anterior descending artery, *CCTA* coronary computed tomography angiography

CCTA is a useful tool for not only stenosis assessment but also plaque assessment. Intravascular ultrasound and optical coherence tomography have been used for plaque assessment, but these techniques require invasive procedures. CCTA allows for noninvasive plaque assessment throughout the coronary trees. In clinical practice, we often identify non-obstructive CAD in CCTA assessment, which is also clinically important. In a meta-analysis, the annual event rate (all-cause or CAD mortality, ACS, or revascularization) in patients with non-obstructive CAD was eight times higher than that in patients without stenosis or plaques [59]. In addition, CCTA could be feasible for the follow-up evaluation of coronary atherosclerotic plaques. Motoyama et al. reported that plaque progression assessed using CCTA was an independent predictor of ACS (HR 33.43, median follow-up period: 4.1 years, median interval period between first CCTA and second CCTA; 1 year, 449 patients) [60]. Lee et al. demonstrated the stabilization of coronary atherosclerotic plaque on CCTA, with decreasing non-calcified components and increasing calcified components after statin therapy (Fig. 11) [61]. With the accumulation of the prognostic significance of coronary atherosclerosis in both obstructive and non-obstructive CAD, the importance of coronary atherosclerosis evaluation using CCTA is expected to increase. The role of CCTA will accordingly expand from only making a diagnosis to leading appropriate treatment strategy of CAD, but further accumulation of evidence is desirable.

### HRP on CCTA

**Fig. 12:**
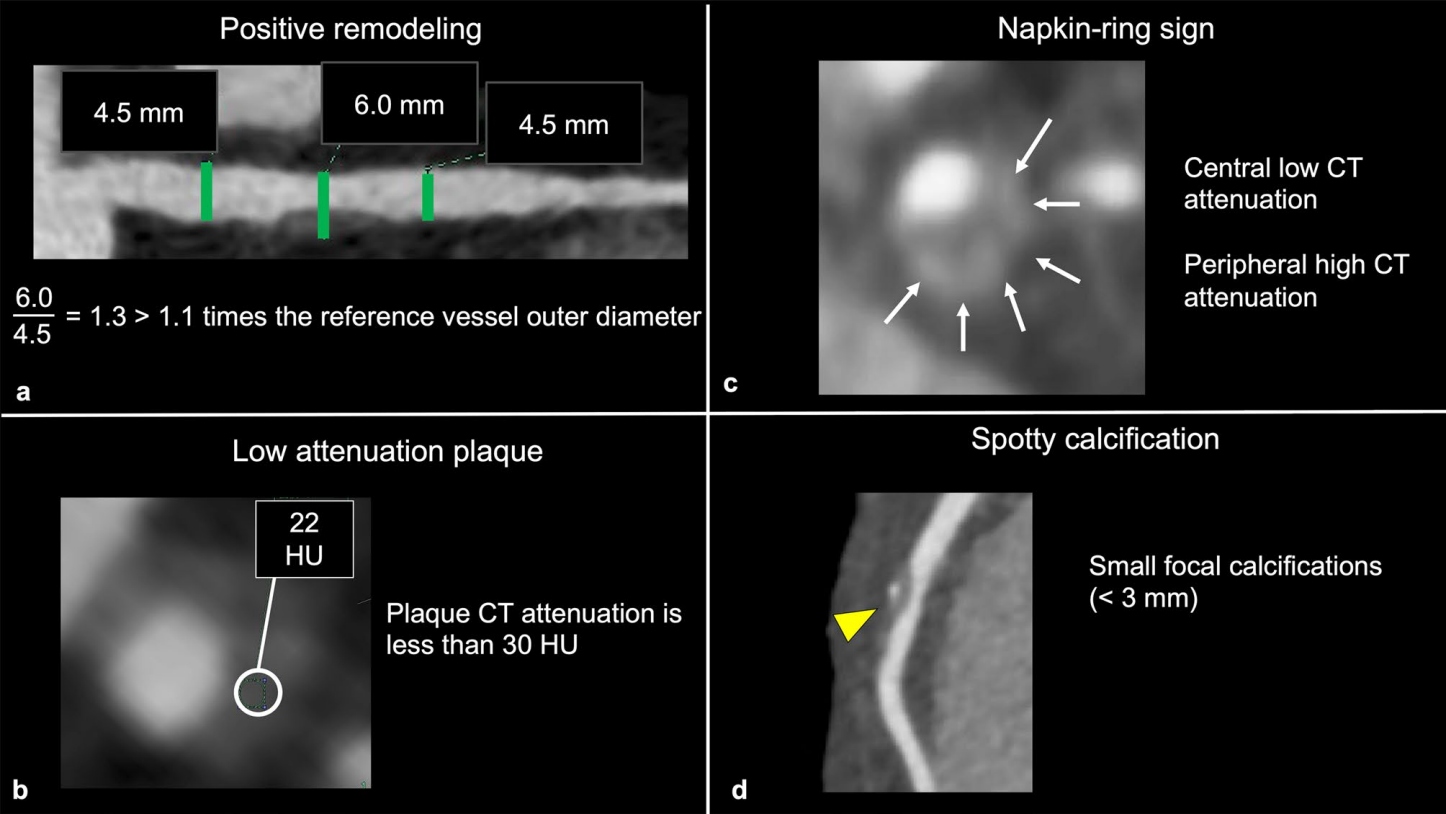
High-risk plaque features on CCTA. Positive remodeling (**a**); the outer diameter at the stenotic lesion is larger than 1.1 times the reference vessel outer diameter. Low-attenuation plaque (**b**); there is a voxel with less than 30 HU in the plaque. Napkin ring sign (**c**); there is a low CT attenuation area of the coronary plaque surrounded by peripheral high attenuation. Spotty calcification (**d**); a small calcification less than 3 mm in diameter is observed in the plaque. The modifier “HRP” should be added in CAD-RADS coding when a coronary plaque has two or more high-risk features on CCTA. *CT* computed tomography, *CCTA* coronary computed tomography angiography, *HRP* high-risk plaque

Vulnerable plaques have histological characteristics such as thin-fibrous cap, necrotic cores, and large plaque volume [62, 63]. Coronary atherosclerotic plaques prone to ACS on CCTA are called “HRP” and have the following features: positive remodeling (PR), low-attenuation plaque (LAP), napkin ring sign (NRS), and spotty calcification (SC) (Fig. 12) [4]. PR is defined as a 10% or more increase in the outer vessel diameter at the plaque site compared to the mean outer diameter of the adjacent normal sites (reference segment) [64, 65]. LAP is defined as a plaque having at least one voxel with CT number < 30 Hounsfield units (HU), corresponding to lipid cores on the pathology [66]. Motoyama et al. reported that coronary atherosclerotic plaques with PR and/or LAP were associated with ACS (HR 22.8, follow-up period: 27 ± 10 months, 1059 patients) [67]. NRS is defined as a central region exhibiting low CT attenuation surrounded by higher attenuation plaque tissue in a ring-like pattern [68]. Coronary atherosclerotic plaques with NRS were also associated with ACS (HR 5.55, follow-up period: 2.3 ± 0.8 years, 895 patients) [69]. SC is defined as the presence of small focal calcifications (< 3 mm in diameter) [59, 70]. In a sub-study of the ROMICAT-II trial, the presence of at least one of the HRP features (PR, LAP, NRS, or SC) was associated with ACS (OR 8.9, follow-up period: 28 days, 472 patients) [71]. In the PROMISE trial, the patients with HRP (defined as PR, LAP, or NRS) had a 70% increased risk of MACE (defined as death, MI, or unstable angina) (follow-up period: median 25 months, 4415 patients) [72]. In the SCOT-HEART trial, patients with HRP (defined as PR and/or LAP) had a threefold higher occurrence of MACE (coronary heart disease death or nonfatal MI) compared to those without HRP (follow-up period: 5 years, 1769 patients) [73]. In CAD-RADS 2.0, it is recommended that the modifier “HRP” is added when a coronary plaque with two or more high-risk features is detected on CCTA [4].

### Pitfall of plaque evaluation on CCTA

**Fig. 13:**
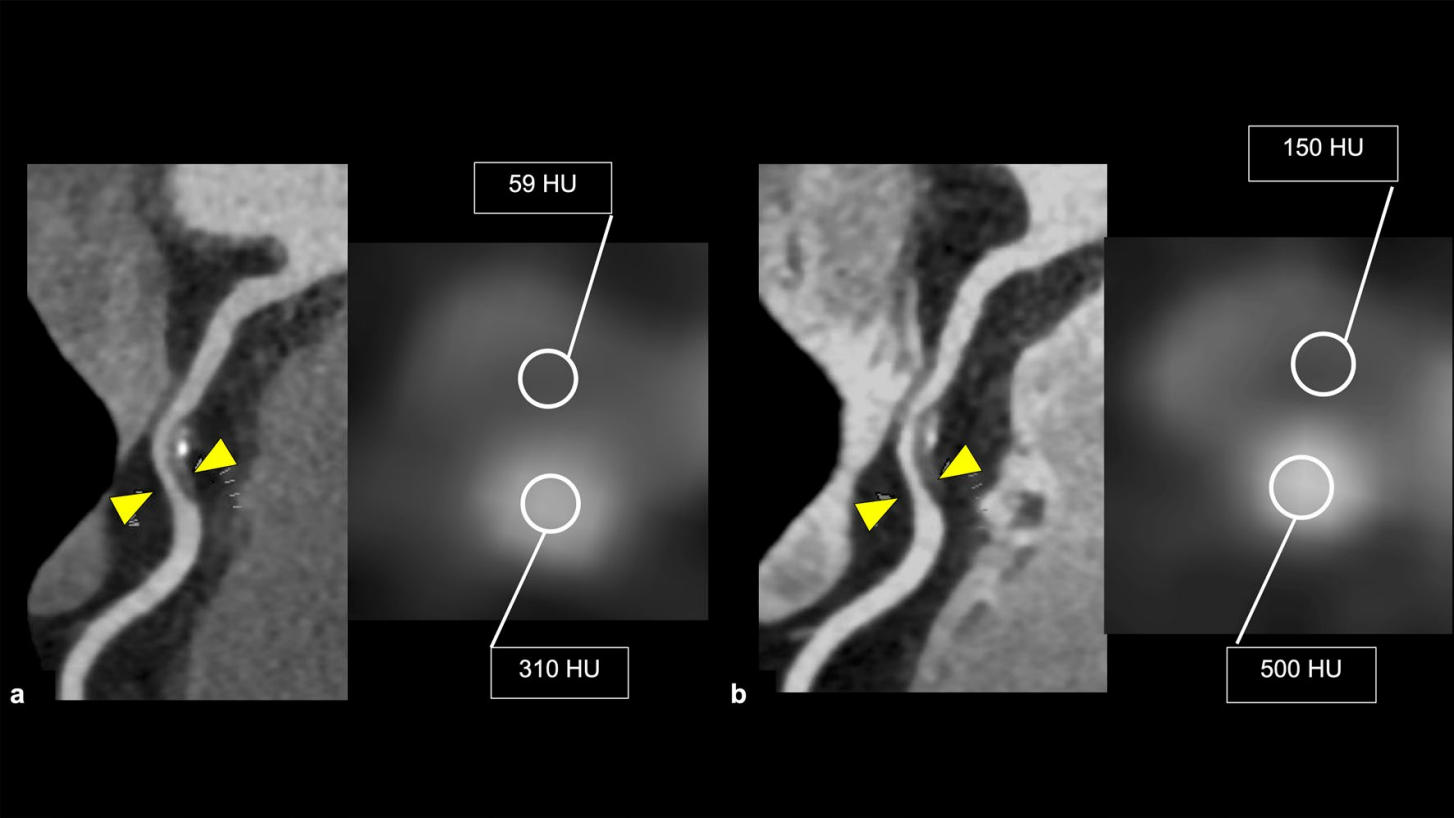
Changes in coronary plaque CT attenuation due to CCTA scanning condition. Two CCTAs are performed in a short interval with the same scan parameters (**a**: first scan, **b**: second scan). CT attenuation of coronary plaque differs between the first and second scans due to the difference in the CT attenuation of coronary artery lumens. *CT* computed tomography, *CCTA* coronary computed tomography angiography

First, the reproducibility of HRP assessment is not high (*κ* = 0.40) [74]. This is partly because qualitative features, such as NRS, are affected by reader experience, and LAP is affected by measurement methods, such as the position and size of the region of interest. Second, tube-voltage setting and intra-coronary attenuation can affect plaque morphology assessment, which should be considered during follow-up CT examination for plaque assessment, especially after medical management (Fig. 13) [75]. Third, HRP on CCTA had relatively low PPV for predicting MACE (4.1–6.4%), despite the relatively high prevalence of HRP on CCTA (15.3–34%) [72, 73]. This may be because the visualization of coronary atherosclerotic plaque on CCTA can encourage the initiation or intensification of medical therapy leading to the stabilization of plaque characteristics [59].

## Findings other than coronary artery stenosis

Radiologists should focus on not only coronary artery findings but also cardiac and extra-cardiac findings such as myocardium, endocardial cavity, valves, and pericardium beyond the coronary artery when reading CCTA images. Moreover, CCTA is increasingly used as a preoperative navigation tool for atrial septal defect (ASD) closure and transcatheter aortic valve implantation (TAVI), and radiologists need to understand the key aspects of CCTA for each procedure. This section will introduce representative cardiac and extra-cardiac findings other than the coronary artery on CCTA and preoperative navigation use.

### Cardiac findings other than the coronary artery

#### Myocardial bridge

**Fig. 14:**
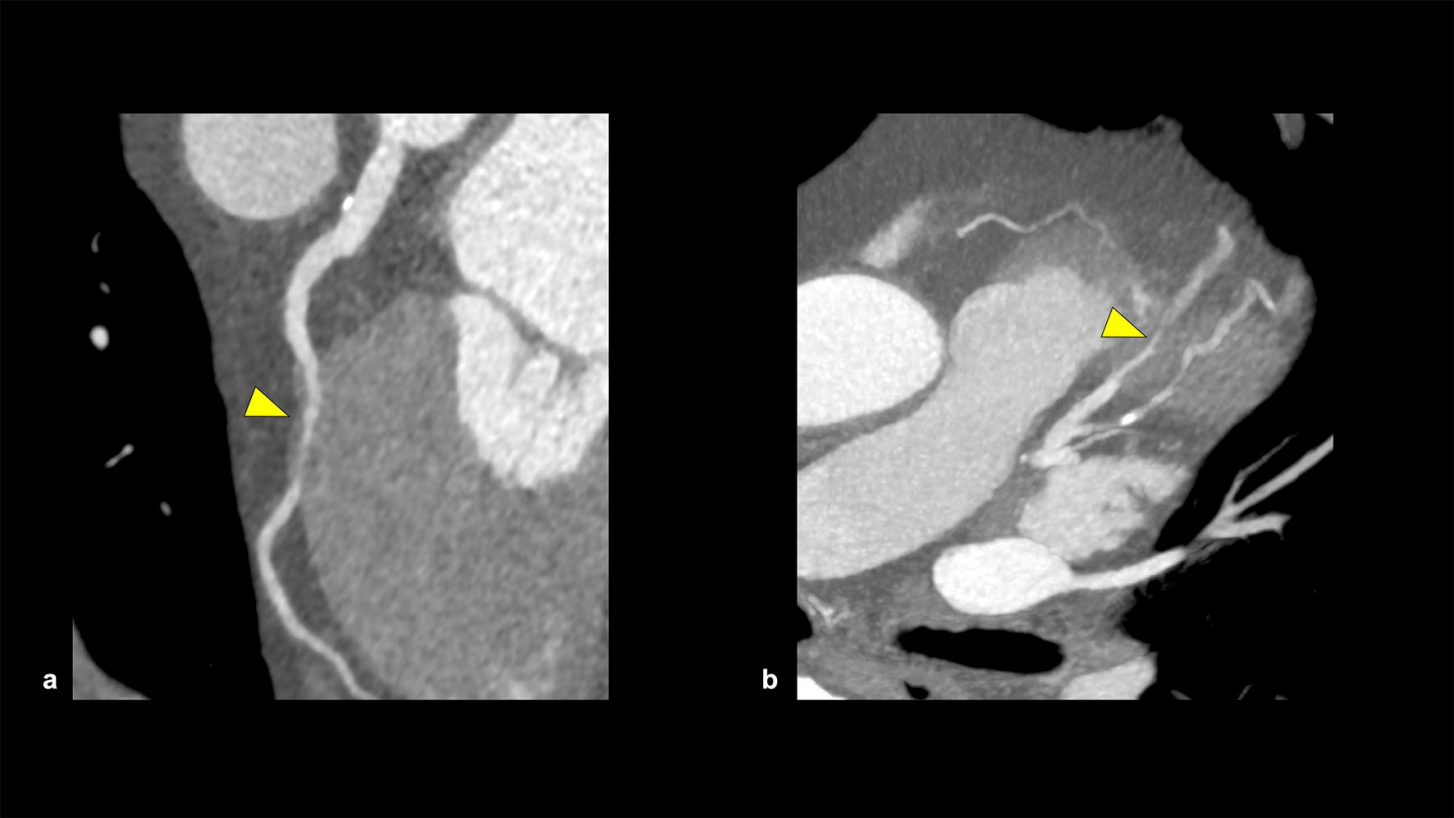
Myocardial bridge. A CPR image shows the myocardial bridge in the mid portion of the LAD (**a**). The coronary artery involved in the myocardial bridge appears stenotic during systole (**b**: MIP). *CPR* curved planar reformation, *LAD* left anterior descending artery, *MIP* maximum intensity projection

Myocardial bridge (MB) is a common coronary artery anomaly and is defined as the coronary artery passing through the myocardium (Fig. 14) [76, 77]. The frequency of MB is 6% on ICA and 22% on CCTA and the left anterior descending (LAD) artery is the most commonly affected region [78]. MB is classified into three types; superficial MB (1–2 mm depth of overlying myocardium), deep MB (≥ 2 mm depth of overlying myocardium), and long MB (≥ 25 mm of overlying myocardium) [76]. Patients with MB are typically asymptomatic, but MB can cause coronary flow obstruction leading to angina or ACS [76].

#### Anomalous aortic origin of a coronary artery

**Fig. 15:**
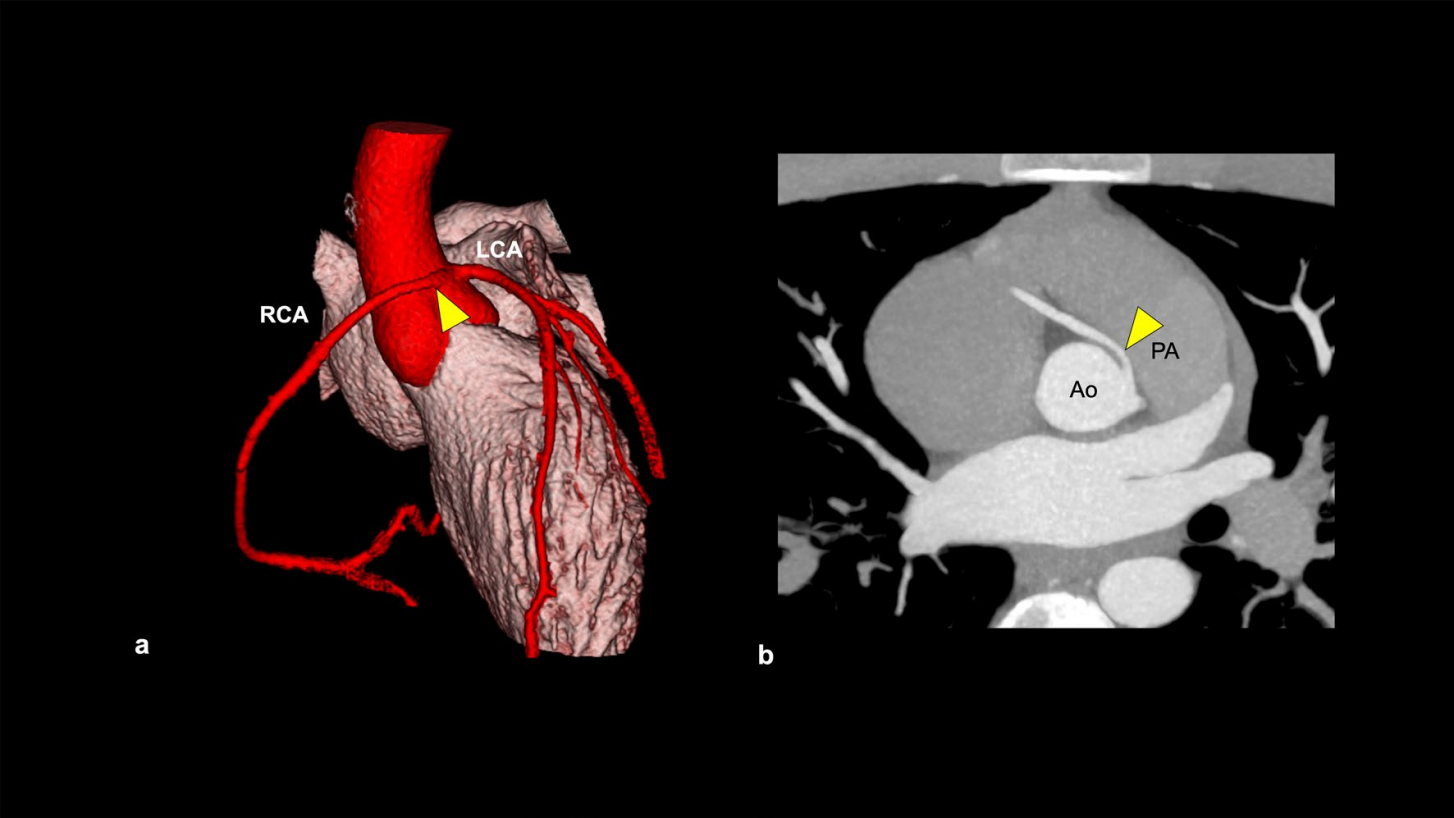
Anomalous aortic origin of a coronary artery. A case of anomalous aortic origin of RCA. CCTA shows that RCA originates from the left aortic sinus of Valsalva and runs between the aorta and pulmonary artery (**a**, **b**). *RCA* right coronary artery, *LCA* left coronary artery, *Ao* aorta, *PA* pulmonary artery

Anomalous aortic origin of a coronary artery (AAOCA) is a rare (0.1–0.7%) coronary artery anomaly (Fig. 15) [77, 79]. AAOCA has multiple variations, which are classified into potentially benign and malignant courses. The potentially malignant courses, such as the inter-arterial course of the left coronary artery, have the risk of causing sudden cardiac death [79].

#### Old myocardial infarction

**Fig. 16:**
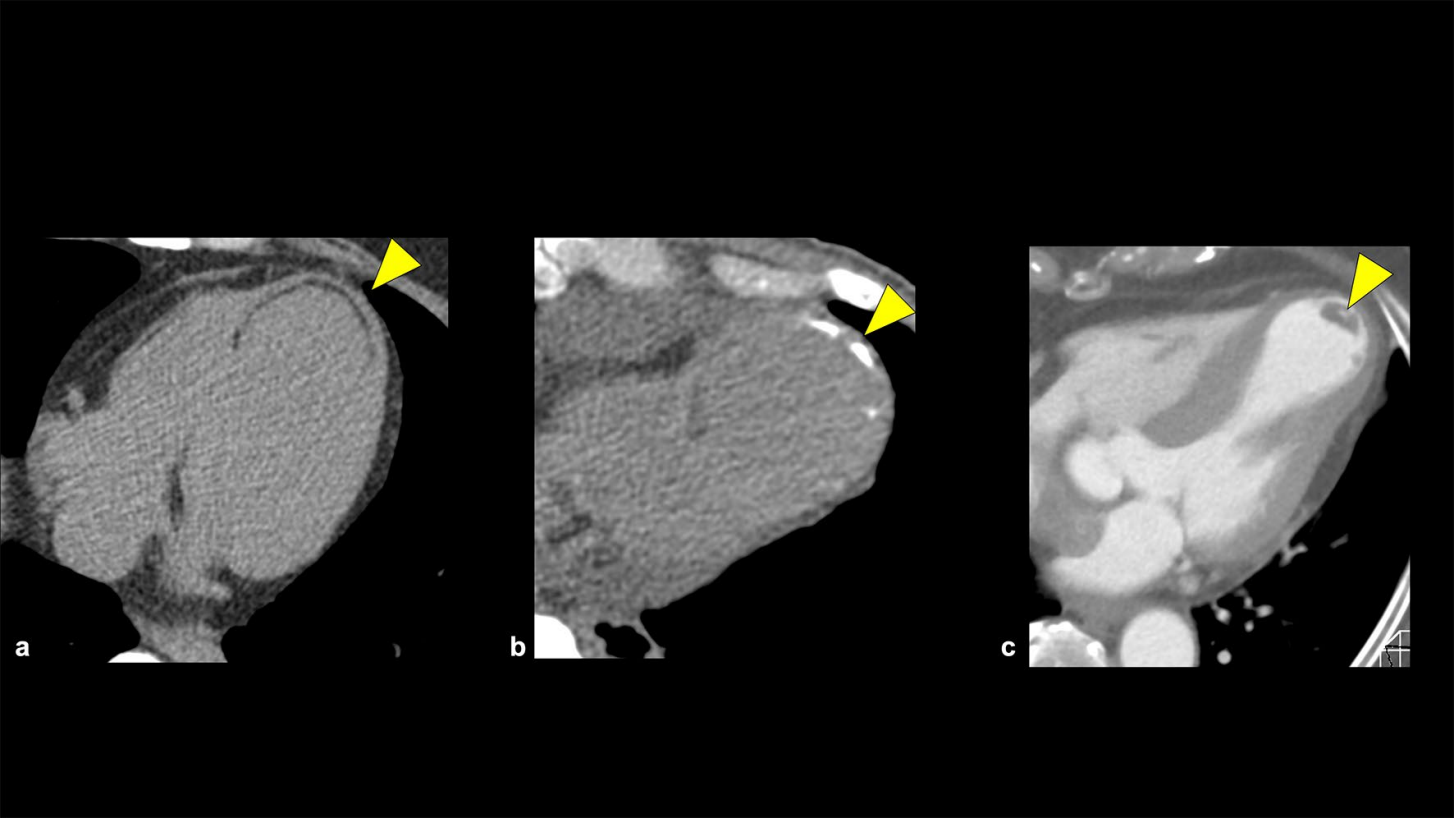
Old myocardial infarction. Old myocardial infarction image shows various CT findings such as fatty degeneration (**a**) and calcification (**b**). Patients with old myocardial infarction have a high risk of intracardiac thrombus (**c**). *CT* computed tomography

Old myocardial infarction (OMI) can be detected using CCTA by observing myocardial changes such as fat deposition, calcification, wall thinning, aneurysmal changes, and perfusion abnormalities (Fig. 16) [80]. OMI becomes more suspicious when these myocardial changes correspond with the perfusion territory of the coronary arteries. Therefore, evaluating these myocardial changes in conjunction with the coronary artery pathway is important. Additionally, intracardiac thrombus should be noted in patients with OMI, since it increases the risk of OMI [80].

#### Patent foramen ovale

**Fig. 17:**
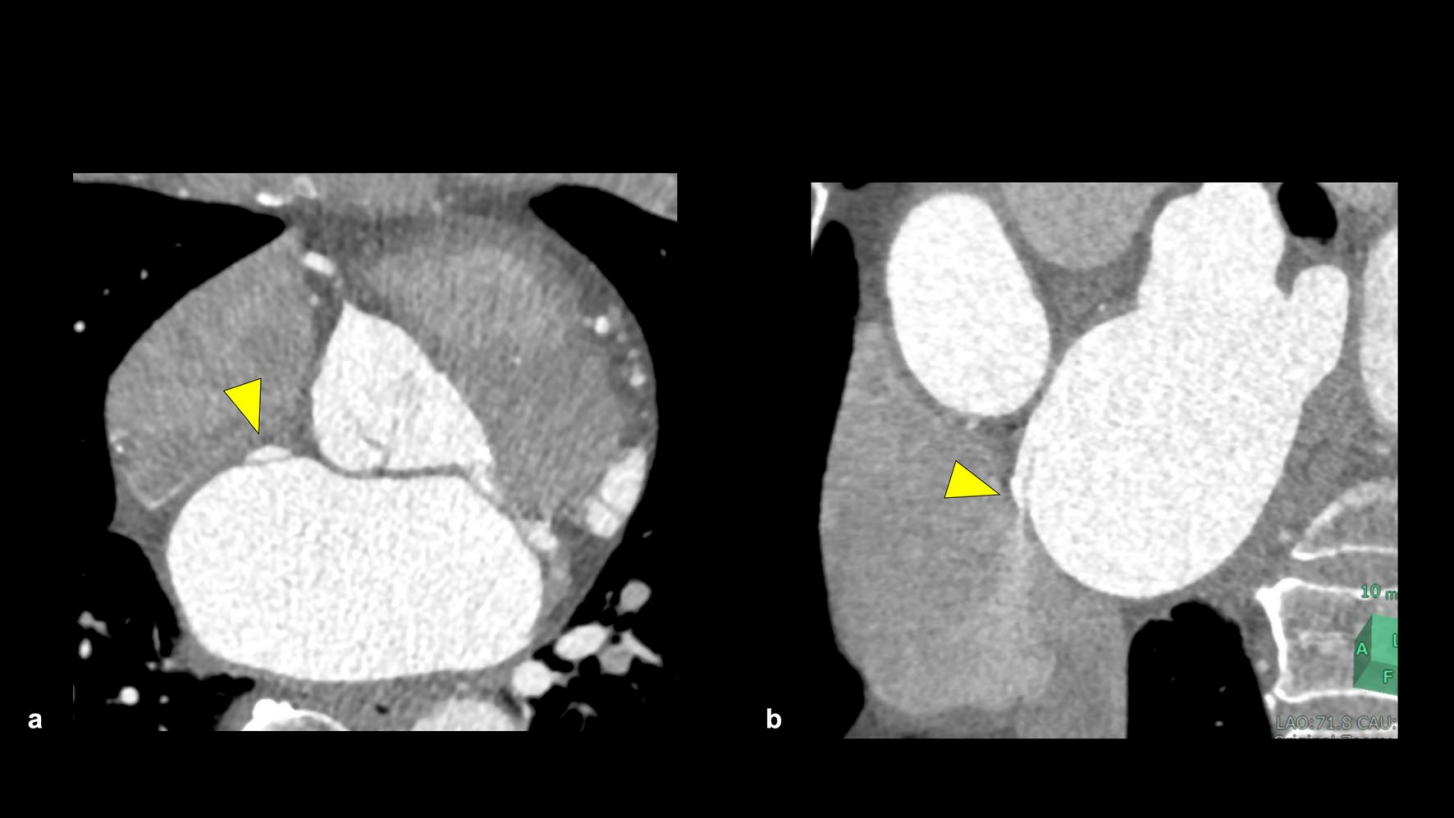
Patent foramen ovale. Axial image shows a left atrial flap (**a**: arrowhead), and coronal images show a contrast agent jet from the left to the right atrium (**b**: arrowhead)

Patent foramen ovale (PFO) is a natural interatrial communication that presents during embryonic circulation from the systemic venous system to the brain (Fig. 17). It persists in approximately 27.3% of humans in autopsy study and is often asymptomatic [81]. However, PFO is at risk of causing cerebral embolism, and PFO closure may be considered in patients with a history of cryptogenic stroke [82]. CCTA can detect PFO using three features: the presence of a left atrial flap in the septum primum, a continuous pathway of contrast material linking the flap in the left atrium to the right atrium, and a jet of contrast material from the column into the right atrium [83].

#### Atrial septal defect

**Fig. 18:**
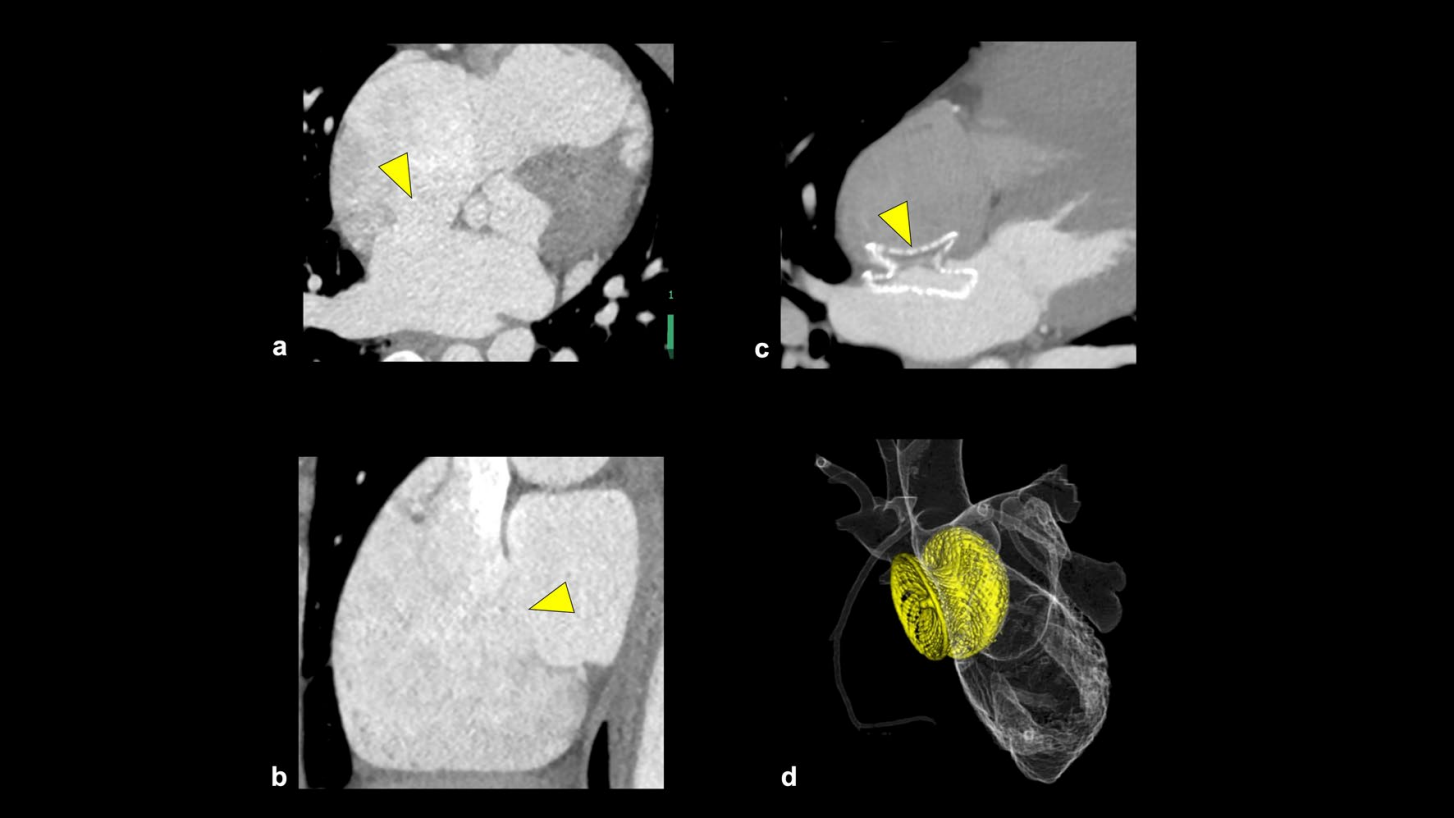
Atrial septal defect. Atrial septal defect (ostium secundum) with an enlarged right atrium is observed on CCTA (**a**, **b**: arrowhead). After transcatheter device closure, CCTA allows for assessing atrial septal defect closure (**c**, **d**). *CCTA* coronary computed tomography angiography

ASD is the most prevalent congenital heart disease among adults with congenital heart disease (Fig. 18) [84]. Based on the defect location, ASD is classified into (1) secundum ASD (80%), (2) primum ASD (15%), (3) superior sinus venosus defect (5%), (4) inferior sinus venosus defect (< 1%), and (5) unroofed coronary sinus (< 1%) [85]. ASD closure is recommended for patients with a significant shunt (as a guide; pulmonary blood flow/systemic blood flow > 1.5) and pulmonary vascular resistance < 5 Wood unit, regardless of symptoms [85, 86]. Device closure is considered for patients with secundum ASD and suitable morphology (defects smaller than 38 mm necessitate a sufficient rim of more than 5 mm except for the anterior margin [85, 86]. In addition, CCTA can assess other cardiovascular malformations such as partial anomalous pulmonary venous return or remnants of the left superior vena cava (common in coronary sinus type) [86].

#### Aortic valve stenosis

**Fig. 19:**
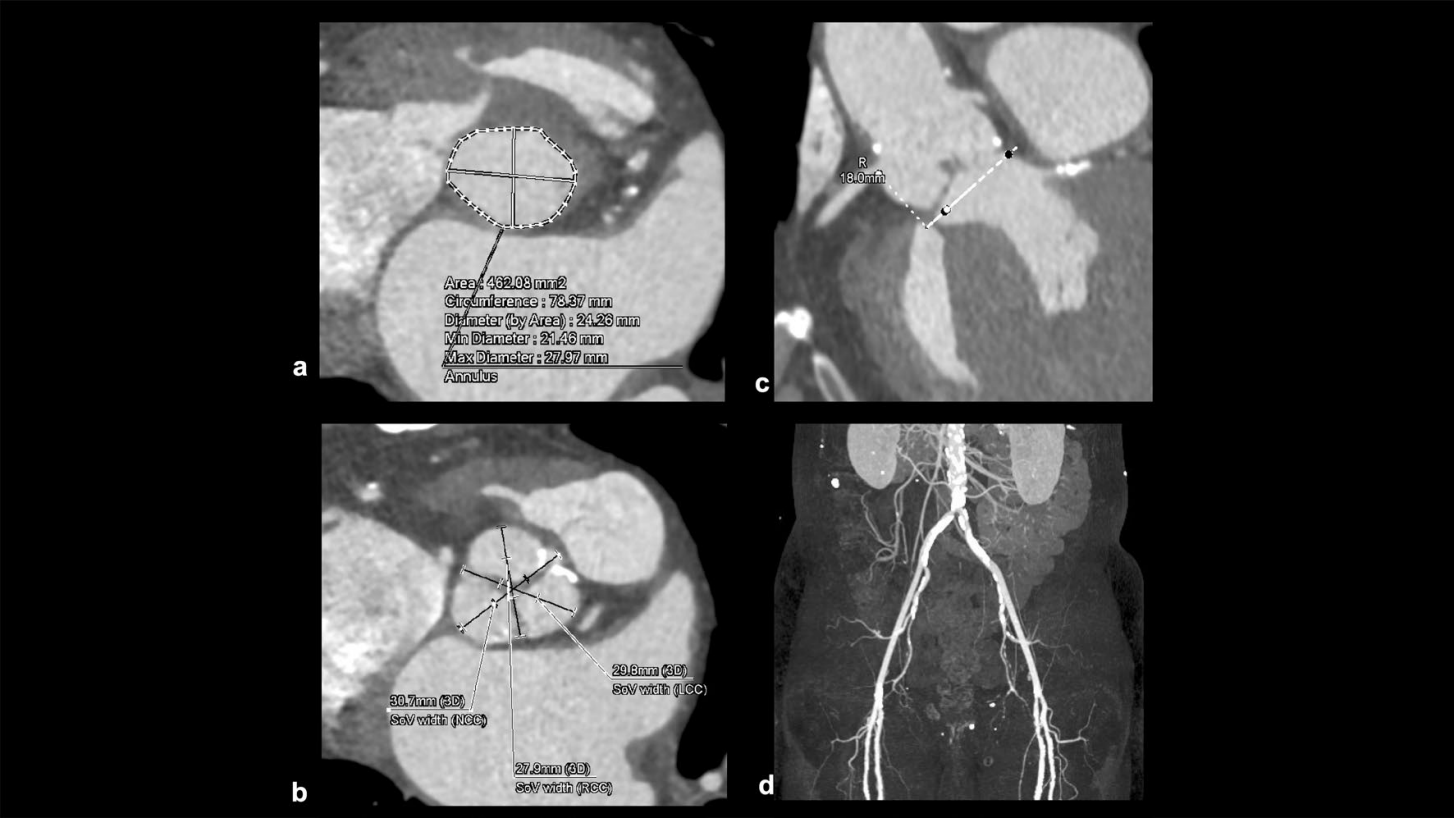
CT imaging for transcatheter aortic valve implantation planning. CT images are used for TAVI planning such as aortic annulus measurement (**a**), sinus of Valsalva measurement (**b**), distance from the coronary ostia to the aortic annulus measurement (**c**), and vascular access route assessment (**d**). *CT* coronary computed tomography, *TAVI* transcatheter aortic valve implantation

The pathophysiology of aortic stenosis (AS) is characterized by chronic pressure overload on the left ventricle due to the narrowing of the aortic valve in the left ventricular outflow tract. Left ventricular hypertrophy and fibrosis occur in response to the increased wall stress caused by this pressure overload [87]. These changes can lead to left ventricular dysfunction, ultimately resulting in hemodynamic instability. Patients with symptoms, such as heart failure, syncope, and chest pain, may die within approximately 2–3 years [88]. Traditionally, surgical aortic valve replacement (SAVR) is the gold standard treatment for patients with severe AS. Recently, TAVI has been developed and has emerged as an alternative treatment strategy for patients with severe AS [87]. JCS stated that the treatment choice between SAVR and TAVI should be based on heart team discussion and patient preferences [87]. CCTA provides essential information on access vessels, aortic root, sinus of Valsalva, aortic annulus, and aortic valve to aid decision-making and TAVI planning (Fig. 19) [89, 90].

#### Left atrial appendage thrombus and slow-flow state

**Fig. 20:**
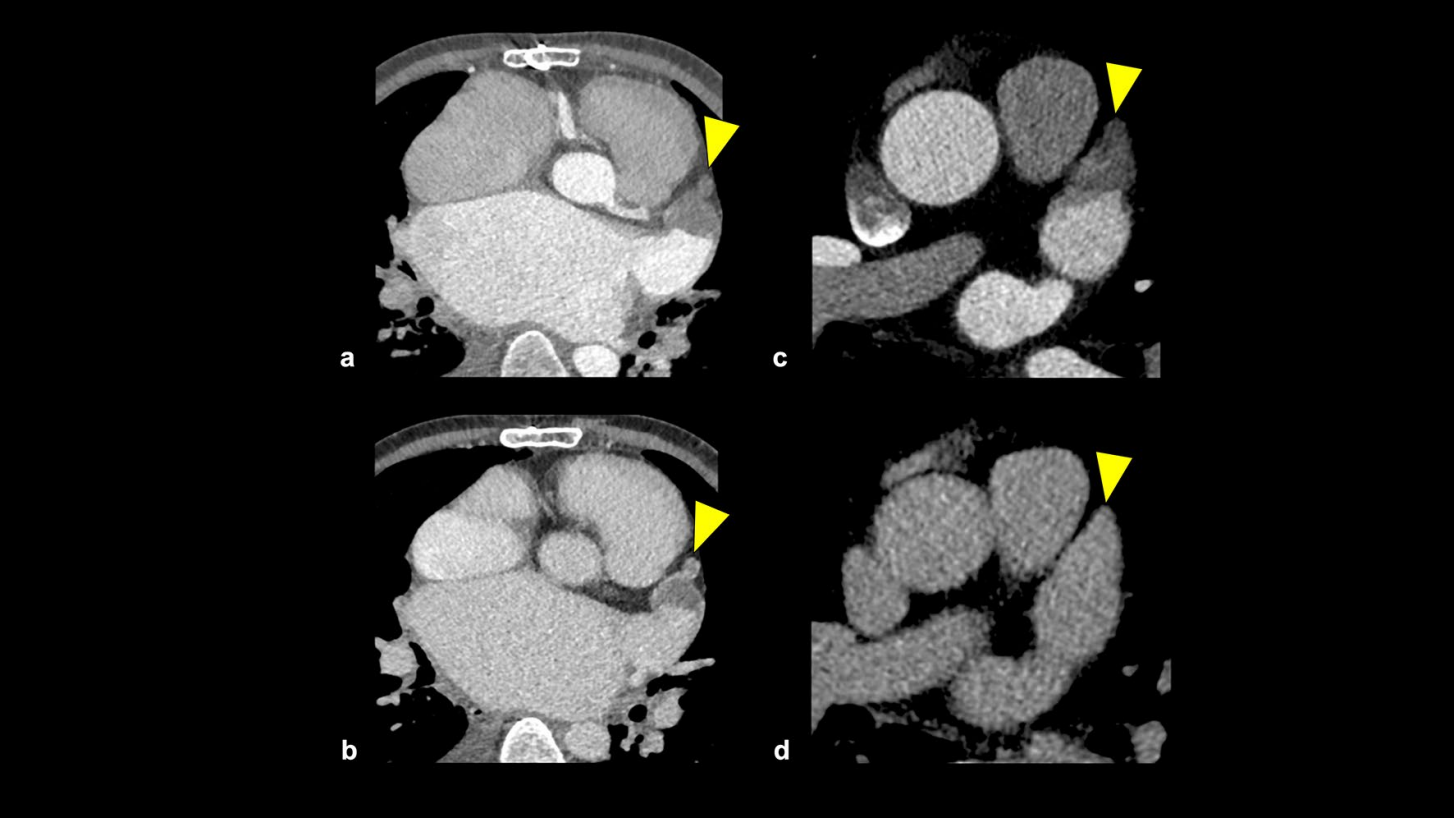
Left atrial appendage thrombus and slow-flow state. Both early-phase and delayed-phase CT images show a contrast defect in the LAA (**a**, **b**: arrowhead), and this case is suspected of the LAA thrombus. Early-phase CT image shows a contrast defect in the LAA (**c**: arrowhead), but the delayed-phase CT image shows no contrast defect in the LAA (**d**: arrowhead). This case is suspected of a slow-flow state. *LAA* left atrial appendage

The left atrial appendage (LAA) is a commonly implicated location for thrombus formation in patients with atrial fibrillation, which can cause cerebral infarction (Fig. 20). LAA thrombus is diagnosed as a contrast-filling defect in CCTA, but this finding can also be observed in patients with the slow-flow state. Delayed image acquisition is useful for distinguishing LAA thrombus and slow-flow state, with a persistent defect suggesting LAA thrombus [91]. CCTA with delayed image acquisitions had a high sensitivity of 100% and specificity of 100% for distinguishing LAA thrombi from circulatory stasis using transesophageal echocardiography as the reference standard [91].

### Extracardiac findings

CCTA sometimes shows incidental findings in the non-cardiac regions. Onuma et al. showed that 58% of patients who underwent CCTA had incidental findings, with 23% of these cases showing significant findings, including 1% of malignancies [92]. Therefore, evaluating all organs in the CCTA scan range is important, and the field of view should be expanded, if necessary, to avoid missing significant incidental findings.

## New technology for CCTA

### Perivascular fat attenuation index (FAI)

FAI was proposed as a novel imaging biomarker that can quantitatively assess coronary inflammation based on the idea that the CT attenuations of peri-coronary adipose tissue increase by inflamed coronary arteries [93, 94]. The FAI is calculated as the average of voxels located between − 190 HU and − 30 HU from the proximal portion of the major coronary arteries such as the right coronary artery (RCA), left anterior descending artery (LAD), and left circumflex artery (LCX) to the 40 mm segments. It should be noted that the RCA is measured from a point 10 mm away from the origin, and the LMT is not included in the measurement. In the post hoc analysis of outcome data of Cardiovascular RISk Prediction using Computed Tomography (CRISP-CT) study, high perivascular FAI values (cutoff ≥ − 70.1 HU) around the proximal RCA could predict cardiac mortality. In addition, several studies demonstrated that FAI was associated with MINOCA [95] and heart failure with preserved ejection fraction [96]. FAI has the potential to diagnose coronary artery inflammation and add incremental value to CCTA.

### Application of AI technology to CCTA

AI has been applied in various aspects of CCTA, such as stenosis, plaque volume assessment, and image reconstruction. Several studies have demonstrated that AI enables rapid assessment of stenosis and plaque volume, showing good agreement with expert readings [97, 98], and can predict future MI [99]. In addition, AI has been applied to image reconstruction leading to effective noise reduction, which is useful for reducing radiation exposure by combining low-dose scans [100, 101]. Recently, a super-resolution deep learning reconstruction technique has been developed, which improves the spatial resolution and sharpness of CCTA images without requiring hardware changes. This method has the potential to improve the detectability of coronary artery stenosis, especially in vessels with small diameters, severe calcification, and stents [54, 55].

### Next-generation CT

We introduce two types of new-generation CT hardware, including ultra-high spatial resolution CT (UHR-CT) and photon-counting detector CT (PCD-CT).

UHR-CT improves spatial resolution from approximately 0.40–0.45 mm to nearly 0.15–0.20 mm [102]. In a coronary artery phantom study (2.0–4.0 mm), UHR-CT demonstrated improved accuracy in assessing coronary artery stenosis compared with conventional CT [103]. Initial human experiences with UHR-CT have demonstrated improved visualization of coronary arteries with calcified plaques or small-diameter stents, which are challenging to evaluate with conventional CT [53]. Furthermore, Takagi et al. reported that UHR-CT improved the quantitative assessment of coronary artery stenosis on CCTA, with a small range of percentage of diameter stenosis (± 16%) using ICA as the reference standard [104].

Conventional energy-integrating detector CT (EID-CT) has solid-state scintillator detector canaries based on indirect conversion technology (two-step). In EID-CT, X-ray photons entering the scintillator generate scintillation light. This scintillation light is then converted into an electrical signal by the photodiode. This electrical signal is amplified, integrated, and serves as the output signal [105]. On the other hand, PCD-CT employs semiconductor detector material based on direct conversion technology (single step). In PCD-CT, X-ray photons entering the detector interact with the detector material and create electron–hole pairs that form a charge cloud. This charge cloud can be directed toward the pixel electrodes through an applied electrical field, resulting in a pulse signal. Ideally, each photon generates a single pulse [105]. PCD-CT offers several advantages over EID-CT, including noise reduction, beam hardening and metal artifacts reduction, spatial resolution improvement, and multi-energy image generation [105, 106]. In fact, in a phantom study focused on coronary stent imaging, PCD-CT enabled the reduction of image noise and stent artifacts, and the improvement of in-stent lumen visibility compared with EID-CT [56]. In an initial human study of CCTA, PCD-CT demonstrated improved image quality and diagnostic confidence in assessing coronary artery stenosis compared with EID-CT [107].

## Conclusions

Radiologists should not only focus on coronary artery stenosis but also coronary atherosclerotic plaque when reading CCTA images, and it is recommended that CCTA reports conform with the CAD-RADS 2.0 guidelines. Additionally, cardiac and extra-cardiac findings other than coronary artery should be noted. FAI can be utilized as an imaging biomarker of coronary inflammation and provide additional information beyond traditional CCTA imaging. AI can be an effective assistant tool when reading CCTA, and next-generation CT technologies offer the potential to overcome the current limitations of CCTA scanned using conventional CT scanners.
